# Engineering *Kluyveromyces marxianus* for 3-hydroxypropionic acid production at elevated temperature from Jerusalem artichoke tubers and crude glycerol

**DOI:** 10.1016/j.synbio.2026.01.008

**Published:** 2026-01-20

**Authors:** Jiacheng Li, Zhongmei Hu, Yanjie Li, Hao Zha, Yujie Xie, Mingtao Zhao, Lili Ren, Biao Zhang

**Affiliations:** a Key Laboratory of Green and Precise Synthetic Chemistry and Applications, Ministry of Education, Huaibei Normal University, Huaibei, Anhui, 235000, PR China; b Anhui Province Key Laboratory of Pollutant Sensitive Materials and Environmental Remediation, School of Life Sciences, Huaibei Normal University, Huaibei, Anhui, 235000, PR China

**Keywords:** *K. marxianus*, 3-Hydroxypropionic acid, Elevated temperature, Jerusalem artichoke tubers, Crude glycerol

## Abstract

This study engineered the thermotolerant yeast *Kluyveromyces marxianus* to produce 3-hydroxypropionic acid (3-HP), a key precursor for biodegradable plastics, via the malonyl-CoA pathway using non-food feedstocks. The 3-HP titer was further increased through deleting *Adh2A* and *Ach1*, which prevents the synthesis of byproducts ethanol and acetic acid. Using Jerusalem artichoke tuber powder, engineered strain produced 27.32 and 32.31 g/L of 3-HP at 37 °C and 42 °C through fed-batch fermentation. Metabolic reconstruction replaced the native FADH_2_-dependent glycerol pathway (GUT1/GUT2) with an NADH-generating GDH1/DAK1 pathway, significantly enhancing glycerol utilization and increasing intracellular NADH supply by 62 %. Overexpression of *Utr1* can further enhance the NADPH supply. Combined with heterologous expression of a codon-optimized, high-activity malonyl-CoA reductase (MCR) mutant (MCR^N940V/K1106W/S1114R^), the engineered strain achieved 3-HP titers of 33.15 g/L in fed-batch fermentation using pure glycerol at 42 °C. Crucially, it also produced 26.57 g/L 3-HP directly from crude glycerol at 42 °C. The thermotolerant fermentation at 42 °C, unprecedented for yeast-based 3-HP synthesis, reduces cooling water consumption by approximately 60 %, translating to an estimated annual CO_2_ reduction of 27.1 tons per 1000-ton fermenter. This work establishes a cost-effective, industrially scalable bioprocess for valorizing Jerusalem artichoke tubers and crude glycerol into a key platform chemical for biodegradable plastics and green chemicals, leveraging the strain's substrate flexibility, process robustness, and significant environmental advantages.

## 1 Introduction

3-Hydroxypropionic acid (3-HP) is an isomer of lactic acid. However, the difference in the relative positions of the hydroxyl and carboxyl groups in its molecular structure endows it with greater chemical versatility. This characteristic makes it a key raw material for the synthesis of bio-based materials and green chemicals [1]. As a versatile organic compound, 3-HP primarily serves as a platform chemical to produce various bio-based chemicals and materials [2,3]. 3-HP can be converted into the polymer poly(3-hydroxypropionic acid) (P3HP), a novel biodegradable plastic [4]. This material exhibits mechanical properties comparable to those of traditional petroleum-based plastics (such as a tensile strength of ≥35 MPa and an elongation at break of >200 %), while also offering the advantages of complete biodegradability and biocompatibility [2,5].

Currently, there are two common methods for synthesizing 3-HP. One method involves traditional chemical synthesis, which utilizes petroleum derivatives (such as ethylene and acrolein) as raw materials to produce 3-HP through catalytic oxidation or hydration reactions [6,7]. Although this method is mature and offers stable yields, it relies on non-renewable resources and involves complex side reactions (such as the formation of toxic by-products like acrolein and acetic acid), leading to environmental pollution and high carbon emissions [8]. This makes it difficult to meet the sustainable development needs under the global carbon neutrality goals. Consequently, the metabolic synthesis of 3-HP using microorganisms has become a hot research topic [6]. At present, there are generally three pathways for microbial production of 3-HP: the glycerol pathway, the malonyl-CoA pathway, and the β-alanine pathway. Based on these three methods, determining which metabolic engineering modifications to implement using raw materials such as methanol, glucose, and glycerol is a research topic worth exploring [6]. However, microbial methods present several challenges. Despite achieving high yields in certain strains, they require stringent control over process parameters such as substrate concentration, temperature, pH, and oxygen supply. Additionally, microbial growth and production processes are susceptible to various environmental factors, which can lead to decreased fermentation performance and productivity of the strains, as well as risks such as contamination during production [2]. The resource consumption and emissions associated with the production process also increase the environmental burden. Therefore, major challenges include discovering robust microbial chassis with high product tolerance and developing efficient fermentation processes that minimize byproduct formation for microbial production of 3-HP [2].

One of the pathways for 3-HP production is the malonyl-CoA pathway, which can utilize glycerol or glucose as substrates for production [6]. Based on our previous research foundation, *K*. *marxianus*, as a novel yeast chassis, exhibits advantages in synthesizing compounds using different carbon sources such as glycerol or inulin, including high growth rates and thermotolerance that other yeasts lack [9]. Therefore, *K*. *marxianus* was constructed for 3-HP production. *K*. *marxianus* is a thermotolerant yeast with great potential for industrial applications [10]. It is the fastest-growing eukaryotic organism and has attracted significant attention in fields such as food, biofuels, enzyme production, and high-value compound synthesis due to its unique physiological characteristics and metabolic flexibility [10]. Its high thermotolerance allows for a significant reduction in the use of cooling water during industrial fermentation processes, thereby saving fermentation costs.

Jerusalem artichoke (*Helianthus tuberosus* L.), with its robust root system, is an exceptionally adaptable perennial herbaceous plant capable of thriving efficiently on marginal lands including arid, cold, saline-alkali, and desertified soils. China's saline-alkali land area reaches 7.67 million hectares, yet large-scale cultivation remains unrealized due to the tuber's high inulin content (over 70 % dry weight)—a non-digestible water-soluble dietary fiber. Humans and monogastric animals (e.g., pigs, chickens) lack enzymes to decompose inulin, preventing direct nutrient absorption and necessitating reliance on gut microbial fermentation for indirect prebiotic benefits, though excessive intake causes bloating or diarrhea. While ruminants can convert inulin into energy via rumen microbes, strict dosage control is essential to avoid disrupting normal fermentation. This characteristic severely diminishes its direct feed value in animal husbandry. *K*. *marxianus*, a microorganism naturally capable of utilizing inulin, has been extensively applied to directly ferment inulin into high-value products like ethanol and lactic acid. Using Jerusalem artichoke tubers (JAT) as fermentation feedstock offers distinct advantages over traditional starch-based materials: its nature as a non-food crop effectively circumvents "competition with human food crops," thereby eliminating upward pressure on grain and feed prices. Its cultivation spatially and functionally complements grain production—enabling industrial feedstock supply from non-arable marginal lands while maintaining existing farmland for food security. Currently, no studies have yet reported 3-HP synthesis through *K*. *marxianus* fermentation of JAT.

In addition, crude glycerol emerges as a significant by-product during the biodiesel manufacturing process. It is approximated that for every 10 kg of biodiesel produced, about 1 kg of crude glycerol is generated [11]. The swift expansion of global biodiesel production in recent years has resulted in a substantial surplus of glycerol, prompting the shutdown of numerous conventional glycerol production facilities. Currently, crude glycerol holds minimal economic value, roughly around $0.1 per kilogram, primarily due to the presence of diverse impurities including methanol, soap, fatty acid methyl esters, and residues of alkaline catalysts [11]. This situation has evolved into a pressing concern and poses financial and environmental burdens for the biodiesel industry. Substantial research efforts have been dedicated to exploring both chemical and biological methods for the value-added transformation of crude glycerol [11,12].

In this study, a *K*. *marxianus* strain capable of synthesizing 3-HP through the malonyl-CoA pathway was constructed through heterologously expressing the malonyl-CoA reductase mutant MCR^N940V/K1106W/S1114R^ from *Chloroflexus aurantiacus*. Knocking out *Adh2A* and *Ach1* prevents the synthesis of byproducts ethanol and acetic acid, further increasing the yield of 3-HP in strain YLJC06. Its 3-HP production efficiency from inulin was significantly enhanced by optimizing oxygen supply and nitrogen source utilization. Ultimately, through fed-batch fermentation in a bioreactor, the engineered strain YLJC06 achieved a 3-HP titer of 27.32 g/L and 32.31 g/L using Jerusalem artichoke tubers as the substrate at 37 °C and 42 °C. Further metabolic engineering enhanced the glycerol metabolic capacity and NADH/NADPH supply of *K*. *marxianus* by reconstructing its glycerol metabolic pathway, switching from the FADH_2_-dependent GUT1/GUT2 pathway to the NADH-dependent GDH1/DAK1 pathway. The resulting strain, heterologously expressing the malonyl-CoA reductase mutant *Ca*MCR^N940V/K1106W/S1114R^, was able to synthesize 15.04 g/L of 3-HP using glycerol, representing an increase of 20.10 % compared to the wild-type strain expressing the *CaMCR*^*N940V/K1106W/S1114R*^ gene. Overexpression of *Utr1* can further increase the content of 3-HP by enhancing NADPH supply in YLJC15.Through fed-batch fermentation in a bioreactor, the strain YLJC15 synthesized 30.95 g/L of 3-HP at 37 °C and 33.15 g/L of 3-HP at 42 °C. Using crude glycerol, it synthesized 22.58 g/L and 26.57 g/L of 3-HP at 37 °C and 42 °C, respectively. This study provides a pathway for the resourceful utilization of low-value crude glycerol, which is prone to environmental pollution.

## 2 Materials and methods

### 2.1 Microorganisms and media

All compounds and biological reagents used in this study meet or exceed analytical grade purity specifications. d-glucose, glycerol, restriction endonucleases (*Eco*RI, *Not*I), T4 DNA ligase, and yeast nitrogen base without amino acids (YNB) were purchased from Sangon Biotech Co., Ltd. (Shanghai, China). *K. marxianus* NBRC1777, a haploid yeast strain sourced from the NITE Biological Resource Center in Tokyo, Japan, served as the foundational strain. In this study, YZB100, a derivative of *K. marxianus* NBRC1777 with a disrupted *Ku70* gene, was utilized as the wild-type reference (Table 1). YZB101, an auxotrophic strain derived from YZB100, harbors a defective *Ura3* gene (Table 1). For the selection of transformants and comparative growth analysis of strains with varying genotypes, YNBD plates were employed. These plates contained 6.7 g/L of yeast nitrogen base (YNB) and 20 g/L of glucose, supplemented with the necessary amino acids. To cultivate *K. marxianus* strains under aerobic or anaerobic conditions, yeast extract-peptone (YP) medium was used, comprising 10 g/L of yeast extract and 20 g/L of peptone, with different carbon sources incorporated as needed. Solid media were prepared by adding 15 g/L of agar to each respective medium. For cloning purposes, *Escherichia coli* XL10-Gold was employed and grown in lysogeny broth (LB) medium.

**Table 1:** Plasmids and strains used in this study.

Plasmids or strains	Relevant genotype	Reference
**Plasmids**		
p414	*Amp*, *Tef1p*-*Cas9*-*Cyc1t*	Addgene 43802
pMD18T-*ΔScUra3*	*AMP*, nonfunctional *ScUra*3	[13]
pZB006	*Amp*, pZB042-*KmGcy1*	This study
pZB007	*Amp*, pZB042-*KmDak1*	This study
pZB023	*Amp*, *Ura3*	[14]
pZB037	*Amp*, pZB023-*KmUtr1*	[15]
pZB075	*Amp*, pUC19-*ScUra3*	[13]
pZB087	*Amp*, pZB023-*OpGdh1*	This study
pZB088	*Amp*, pZB023-*CjFps1*	This study
pZB089	*Amp*, pUC19-*Snr52p*	[16]
pZB090	*Amp*, pUC19-gRNA.CAN1.Y-*Sup4t*-*Cyc1t*	[16]
pZB211	Amp, *P*_*ScSnr52*_-DR	[17]
pZB212	Amp, DR*-T*_*ScCyc1*_	[17]
pZB216	*Amp*, pZB023-*MCR*^*N940V/K1106W/S1114R*^	This study
pZB273	*Amp*, *P*_*KmPdc1*-_Cas12a-T_*ScCyc1*_	[17]
**Strains**		
*K. marxianus* NBRC1777	Wild type, from NBRC	NBRC
YZB100	YZB040, *ΔKu70*::*Ura3*	[16]
YZB101	YZB100, *ΔUra3*	[16]
YZB154	YZB100, *ΔGut2*	[15]
YZB177	YZB100, *ΔGut1ΔGut2*	[15]
YZB195	YZB100, *ΔGut1ΔGut2ΔGcy1*	[15]
YZB196	YZB195, *ΔUra3*	[15]
YZB401	YZB101, *SpCas9*	This study
YZB402	YZB401, *ΔUra3*	This study
YLJC01	YZB402, *MCR*^*N940V/K1106W/S1114R*^	This study
YLJC02	YLJC01, *ΔUra3*	This study
YLJC03	YLJC02, *ΔAdh2A*	This study
YLJC04	YLJC02, *ΔAch1*	This study
YLJC05	YLJC03, *ΔUra3*	This study
YLJC06	YLJC05, *ΔAch1*	This study
YLJC07	YZB196, *OpGdh1*	This study
YLJC08	YLJC07, *ΔUra3*	This study
YLJC09	YLJC08, *CjFps1*	This study
YLJC10	YLJC09, *ΔUra3*	This study
YLJC11	YLJC10, *KmDak1*	This study
YLJC12	YLJC11, *ΔUra3*	This study
YLJC13	YLJC12, *MCR*^*N940V/K1106W/S1114R*^	This study
YLJC14	YLJC13, *ΔUra3*	This study
YLJC15	YLJC14, *Utr1*	This study
YLJC16	YLJC14, *Gcy1*	This study

### 2.2 Plasmids construction

Table 1 provides a summary of the plasmids and strains used in this study. The primers utilized in this research, which were purchased from General Biotech Co. (Chuzhou, China), are listed in the supplementary material (Table S1). The plasmid p414 was obtained from Addgene (Watertown, MA, USA). For the amplification of the Snr52 promoter, pZB089 was used, while pZB090 was employed to amplify the gRNA.CAN1.Y-Sup4t-Cyc1t fragment. The plasmid pMD18T-*ΔScUra3* was utilized to amplify the fragment of a truncated-*ScURA3*, which served as the genetic selection marker for regeneration (Table 1) [13]. *KmGcy1* and *KmDak1* was inserted into pZJ042 [18] at the *Eco*RI and *Not*I to generate pZB006 and pZB007. pZB037 includes a *KmUtr1* expression cassette [15]. *Gdh1* from *Ogataea parapolymorpha*, *Fps1* from *Cyberlindnera jadinii* and *MCR*^N940V/K1106W/S1114R^ from *C. aurantiacus* were was codon-optimized and synthesized by General Biotech Co. (Chuzhou, China). After that, *OpGdh1*, *CjPfs1*, and *CaMCR*^N940V/K1106W/S1114R^ were inserted into pZB023 to generate pZB087, pZB088, and pZB216. (Table 1). pZB211 and pZB212, containing the fragment *P*_*ScSnr52*_-DR and DR-*T*_*ScCyc1*_, respectively, served as templates for constructing crRNA for CRISPR/Cas12a using fusion PCR [17]. pZB273 containing a *FnCas12a* expression cassette controlled by *P*_*KmPdc1*_ [17].

### 2.3 Construction of 3-HP production strain of *K. marxianus* through malonyl-CoA pathway

To facilitate gene editing in *K*. *marxianus*, the *Cas9* gene expression cassette was first integrated into the *Xyl1* gene locus of the YZB101 genome (Fig. S1A). Using the transient CRISPR/Cas9 method, the *Cas9* expression cassette from p414 and the *Ura3* gene expression cassette from pZB023 were initially amplified, these two fragments shared a 60 bp overlapping region (Fig. S1B). Subsequently, the transcription cassette of the sgRNA targeting the *Xyl1* gene was amplified through overlap extension PCR (Fig. S1C and D). The three fragments were mixed and subjected to ethanol precipitation, followed by transformation into YZB101 cells using the lithium acetate method [19]. The resulting transformants underwent genomic DNA extraction and PCR verification, yielding the recombinant strain YZB401 with the integrated *Cas9* gene (Fig. S1E). For further genetic manipulation, the *Ura3* gene in YZB401 was knocked out, resulting in strain YZB402.

The mutant *MCR*^*N940V/K1106W/S1114R*^ of *CaMCR* has been reported to exhibit enhanced activity compared to the wild-type. Therefore, we synthesized the *MCR*^*N940V/K1106W/S1114R*^ mutant gene. The *MCR*^*N940V/K1106W/S1114R*^ mutant gene expression cassette was amplified from pZB216 with primers LAC4-M13-F/LAC4-M13-R and integrated into the *Lac4* gene locus of *K. marxianus* YZB402 using transient CRISPR/Cas9 technology, resulting in the strain YLJC01. The *Lac4* gene encodes β-galactosidase, and the deletion of this gene has no significant impact on the normal growth of *K*. *marxianus.* In order to reduce ethanol production during 3-HP fermentation, *Adh2A*, previously identified as the primary alcohol dehydrogenase responsible for ethanol synthesis in *K*. *marxianus* [17], was deleted to minimize ethanol formation, resulting in strain YLJC03. Meanwhile, *Ach1*, the key gene involved in acetate synthesis, was knocked out to prevent acetate production, yielding strain YLJC04. Following the deletion of the *Ura3* gene in YLJC03, strain YLJC05 was obtained. Subsequently, the *Ach1* gene was also knocked out in this strain to generate YLJC06, which represents the dual knockout strain lacking both *Adh2A* and *Ach1*.

### 2.4 Testing of an engineered *K*. *marxianus* strain for 3-HP synthesis via the malonyl-CoA pathway

The wild-type strain and engineered strains YLJC01, YLJC03 YLJC04, and YLJC06 was inoculated into YPD medium for pre-culture at 37 °C. After overnight growth, the culture was transferred to fermentation media including YPD, YPF (YP medium with 2 % fructose), YPI (YP medium with 2 % inulin), and YPG (YP medium with 2 % glycerol), and fermented at 30 °C, 37 °C, 42 °C or 45 °C. Upon completion of fermentation (36 h for YPD, YPF, and YPI, whereas it was 84 h for YPG), the supernatant was collected and analyzed for 3-HP concentration using HPLC. The experiments were conducted at 37 °C with a shaking speed of 220 rpm.

Subsequently, the growth curves, substrate utilization, 3-HP titer, and concentrations of byproducts ethanol and acetic acid were measured for YLJC01, YLJC03 YLJC04, and YLJC06 utilizing glucose, fructose, and inulin to synthesize 3-HP at 30 °C, 37 °C, 42 °C, and 45 °C. Furthermore, the strain's capability to produce 3-HP from YP medium with 50, 100, 150, and 200 g/L Jerusalem artichoke tubers powder was evaluated with fermentation time of 48 h, 72 h, 108 h, and 132 h, alongside the impact of oxygen supply on 3-HP synthesis. Ulteriorly, comparative analyses of 3-HP yields from YP medium with 100 g/L Jerusalem artichoke tubers powder were conducted under different fermentation conditions: flasks sealed with sealing film (F + S), flasks wrapped with gauze (F + G), baffled flasks sealed with sealing film (B + S), and baffled flasks wrapped with gauze (B + G), the fermentation time were 72 h. The effects of various industrial nitrogen sources (soybean peptone (SP), fish meal peptone (FP), corn steep liquor (CL), and soybean meal extract (SE)) on 3-HP production from inulin by YLJC06 were compared using baffled flasks wrapped with gauze, with 100 g/L Jerusalem artichoke tubers powder and 30 g/L each industrial nitrogen sources plus 5 g/L (NH_4_)_2_SO_4_ as nitrogen source, the fermentation time were 72 h. Finally, the effect of CaCO_3_ on 3-HP synthesis by YLJC06 were tested using 30 g/L fish meal peptone with 100 g/L Jerusalem artichoke tubers powder, supplementing with 25 g/L, 30 g/L, 35 g/L, and 40 g/L CaCO_3_, and conducted fermentation in baffled flasks wrapped with gauze at 37 °C, the fermentation time were 72 h.

### 2.5 YLJC06 utilized batch and fed-batch fermentation in bioreactors to synthesize 3-HP from Jerusalem artichoke tubers powder

3-HP synthesized from Jerusalem artichoke powder by strain YLJC06 in fermenters at 37 °C and 42 °C was tested. Fermentation conditions were set as follows: temperature at 37 °C or 42 °C, aeration rate of 1 vvm, agitation speed of 450 rpm, initial substrate concentration of 100 g/L Jerusalem artichoke tuber powder, and 30 g/L fish meal peptone as nitrogen source. Additionally, 35 g/L CaCO_3_ were added to comparison the 3-HP titers between batch and fed-batch fermentation processes at 42 °C. The conditions for fed-batch fermentation are as follows: 100 g/L Jerusalem artichoke tuber powder with 30 g/L fish meal peptone at 42 °C, an aeration rate of 1 vvm, and the addition of 35 g/L CaCO_3_; after the Jerusalem artichoke tuber powder was depleted, supplement with an additional 100 g/L Jerusalem artichoke tuber powder and 30 g/L fish meal peptone. In fed-batch fermentations with Jerusalem artichoke tuber hydrolysate, feeding was initiated based on reducing sugar concentration, maintaining it above a threshold of 10 g/L to support continuous production.

### 2.6 Re-construction of glycerol metabolism pathway in *K. marxianus*

Building upon our previously constructed triple-gene deletion strain *gut1Δgut2Δgcy1Δ* (YZB195), and its *Ura3* deleted strain YZB196 [15]. Using strain YZB196 as a foundation, we employed transient CRISPR/Cas9 technology to integrate the *OpGdh1* gene into its original *Ura3* locus, yielding strain YLJC07. Subsequently, the *Ura3* gene was knocked out in strain YLJC07 to obtain strain YLJC08. Furthermore, the *CjFps1* gene was integrated into the *Xyl2* locus of strain YLJC08, generating strain YLJC09. The *Ura3* gene was then knocked out in strain YLJC09, resulting in strain YLJC10. Next, the *KmDak1* gene was integrated into the *Xyl1* locus of strain YLJC10, producing strain YLJC11. The *Ura3* gene was knocked out in strain YLJC11 to obtain strain YLJC12. Subsequently, the *MCR* ^*N940V/K1106W/S1114R*^ gene was integrated into the *Lac4-1* locus of strain YLJC12, yielding strain YLJC13. The *Ura3* gene was knocked out in strain YLJC13to obtain strain YLJC14. Finally, the *KmUtr1* or *KmGcy1* expression cassette was integrated into the *Lac4-2* locus of strain YLJC14, yielding strain YLJC15 and YLJC16, respectively.

### 2.7 Determination of glycerol metabolic capacity in glycerol-reconstructed strains

Different strains undergoing glycerol metabolic pathway reconstruction and the control strain YZB100 were pre-cultured in YPD medium and then transferred to 250 mL Erlenmeyer flasks containing 50 mL of YNB + 2 % glycerol medium for cultivation. The OD values of the strains were measured at different time points. The experiments were conducted at 37 °C with a shaking speed of 220 rpm.

### 2.8 Testing of intracellular DHA content in glycerol-reconstructed strains

Different strains undergoing glycerol metabolic pathway reconstruction and the control strain YZB100 were pre-cultured in YPD medium and then transferred to 250 mL Erlenmeyer flasks containing 50 mL of YPG medium for cultivation until the logarithmic phase. The cell pellet was collected by centrifugation and washed three times with sterile water. The cells were disrupted using a pressure homogenizer, and the supernatant was collected after centrifugation. The intracellular dihydroxyacetone (DHA) concentration was determined using HPLC with a refractive index detector.

### 2.9 Testing of intracellular NADH and NADPH content in glycerol-reconstructed strains

Strains YZB100, YZB154, YZB177, YZB195, YLJC07, YLJC09, YLJC11, YLJC13, YLJC15, and YLJC16 were pre-cultured in YPD medium and then transferred to 250 mL Erlenmeyer flasks containing 50 mL of YPG medium for cultivation until the logarithmic phase. The cell pellet was collected by centrifugation and washed three times with sterile water. The cells were disrupted using a pressure homogenizer, and the supernatant was collected after centrifugation. The intracellular NADH and NADPH content in yeast cells was measured using a detection kit, following the method previously reported [18].

### 2.10 Comparison of the ability of YLJC01, YLJC13, YLJC15, and YLJC16 to synthesize 3-HP from glycerol

Strains YLJC01, YLJC13, YLJC15, and YLJC16 were inoculated into YPD medium for pre-culture. After overnight growth, they were transferred to YPG medium. Upon completion of fermentation, the supernatant was collected and analyzed for 3-HP concentration using HPLC. The experiments were conducted at 37 °C with a shaking speed of 220 rpm.

### 2.11 Batch and fed-batch fermentation of YLJC15 using glycerol in a fermenter

For batch fermentation, YLJC15 was pre-cultured in YPD medium to the logarithmic phase and then inoculated at 10 % (V/V) into a 5 L fermenter with a working volume of 2.5 L. The fermentation medium consisted of YPG supplemented with 35 g/L CaCO_3_, and the fermentation was conducted at 37 °C with an agitation speed of 450 rpm and an aeration rate of 1 vvm. During fed-batch fermentation, YLJC15 was firstly fermented using the same conditions as batch, when most of the glycerol was consumed, glycerol was fed into the fermenter. Similarly, fed-batch fermentation was carried out at 42 °C under the same conditions.

### 2.12 Fed-batch fermentation of YLJC15 using crude glycerol in a fermenter

YLJC15 was pre-cultured in YPD medium to the logarithmic phase and then inoculated as a 10 % (V/V) inoculum into a 5 L fermenter with a working volume of 2.5 L. The fermentation medium was YPG with 35 g/L CaCO_3_, and the glycerol used was derived from biodiesel by-products. The fermentation was conducted at both 37 °C and 42 °C with an agitation speed of 450 rpm and an aeration rate of 1 vvm. When most of the glycerol was consumed, crude glycerol was supplemented into the fermenter.

### 2.13 Analytical methods

Glucose, fructose, inulin, glycerol, and DHA were separated using a ROA-Organic Acid H^+^ (8 %) column (Phenomenex, Torrance, Los Angeles, California, USA) at 60 °C using a refractive index detector and an injection volume of 20 μL as previously described [20] while 3-HP was detected using an ultraviolet detector with a detection wavelength of 210 nm.

## 3 Results and discussion

### 3.1 Construction of a malonyl-CoA pathway in *K. marxianus* for 3-HP synthesis

**Fig. 1:**
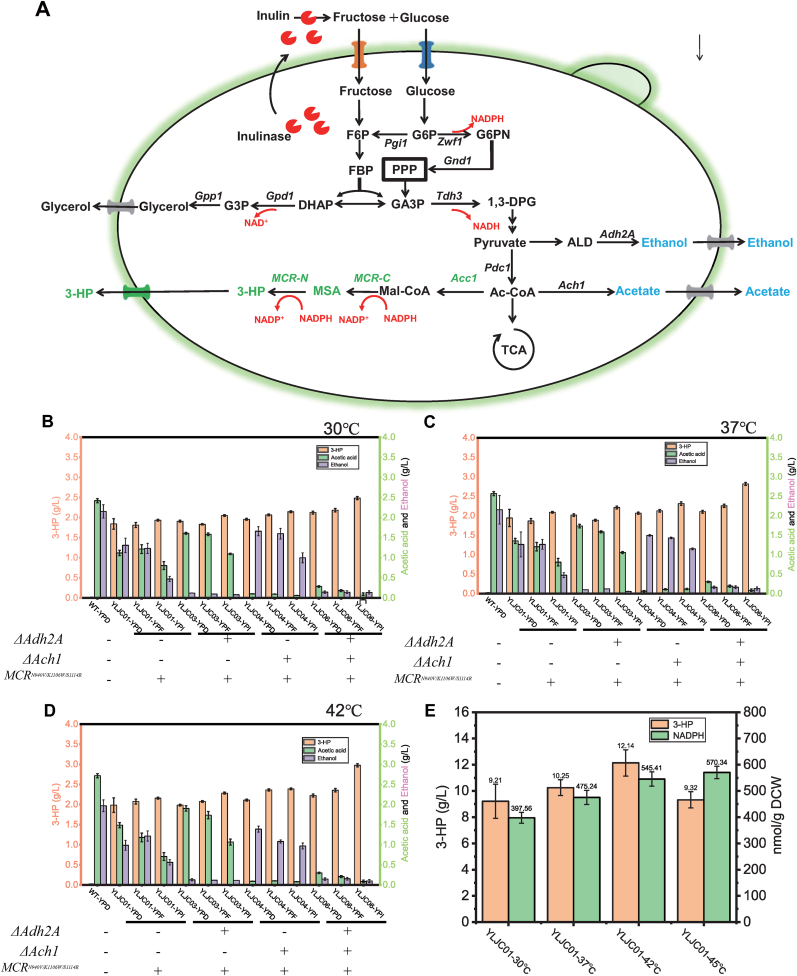
(A) The schematic diagram of the malonyl-CoA pathway for 3-HP synthesis from glucose, fructose, and inulin. The performance of strains YZB401, YLJC01, YLJC03, YLJC04, and YLJC06 in 3-HP synthesis using glucose, fructose, and inulin at 30 °C (B), 37 °C (C), and 42 °C (D). (E) YLJC01 utilizing YPG for the synthesis of 3-HP at 30 °C, 37 °C, 42 °C, and 45 °C. Data are shown as the mean ± SD from at least three experiments.

The malonyl-CoA reductase mutant MCR^N940V/K1106W/S1114R^ from *C*. *aurantiacus* has been extensively demonstrated to possess a high capacity for synthesizing 3-HP from malonyl-CoA. Its C-terminal (MCR-C) and N-terminal (MCR-N) domains are responsible for different reactions: the C-terminal domain catalyzes the synthesis of malonate semialdehyde (MSA) from malonyl-CoA, while the N-terminal domain catalyzes the synthesis of 3-HP from MSA (Fig. 1A). Therefore, some studies have suggested that separating the expression of the C-terminal and N-terminal domains of malonyl-CoA reductase and adjusting their expression levels differently can enhance 3-HP synthesis [21]. However, other studies have indicated that fused expression of MCR-C and MCR-N is more conducive to 3-HP synthesis [22]. In this study, the fused expression of the malonyl-CoA reductase mutant *MCR*^*N940V/K1106W/S1114R*^ was used. The *MCR*^*N940V/K1106W/S1114R*^ gene from *C*. *aurantiacus* was codon-optimized and synthesized, placed under the control of the strong constitutive promoter of *Pgk1* of *K*. *marxianus* and obtained the plasmid pZB216. Then, using transient CRISPR/Cas9, the *MCR*^*N940V/K1106W/S1114R*^ expression cassette was integrated into the upstream of lactose metabolism gene *Lac4* locus of *K*. *marxianus* YZB402, yielding strain YLJC01.

To assess the functionality of MCR^N940V/K1106W/S1114R^ in *K*. *marxianus,* the 3-HP synthesis capabilities of strains YLJC01 and YZB401 were examined using YPD medium at 30 °C, 37 °C and 42 °C. As depicted in Fig. 1B–D and Fig. S2, strain YLJC01 transformed with *MCR*^*N940V/K1106W/S1114R*^ produced 1.84 g/L, 1.94 g/L, 1.98 g/L, and 1.52 g/L of 3-HP, whereas YZB401 showed nearly undetectable levels of 3-HP (Fig. 1B–D, and Fig. S2). These results indicate that MCR^N940V/K1106W/S1114R^ can function in *K*. *marxianus* to mediate the synthesis of 3-HP. However, during this process, when strain YLJC01 synthesized 3-HP using YPD medium at 30 °C, 37 °C, 42 °C and 45 °C, it still produced ethanol as by-products at levels of 1.31 g/L, 1.26 g/L, 0.99 g/L, and 1.21 g/L respectively, along with acetate at 1.12 g/L, 1.35 g/L, 1.48 g/L, and 1.64 g/L respectively. The ability of YLJC01 to synthesize 3-HP using fructose and inulin as carbon sources was further tested. As shown in Fig. 1B–D at different temperatures, the 3-HP production by YLJC01 using fructose was similar to that with glucose. However, when inulin was used, the 3-HP yield increased by 5 %–9 % compared to glucose (Fig. 1B–D), suggesting that a slower substrate metabolism rate may help reduce by-product formation and improve 3-HP production (with inulin as the carbon source, by-product levels decreased by 25 %–60 % compared to glucose and fructose) (Fig. 1B–D). This is because *K. marxianus* must first secrete inulinase to hydrolyze inulin into glucose and fructose during utilization, thereby slowing the metabolic rate. Consequently, more carbon source is directed toward biomass growth, reducing by-product formation and enhancing 3-HP synthesis (Fig. 1A) [14]. Therefore, we hypothesize that knocking out *Adh2A* and *Ach1* in *K*. *marxianus* to block the synthesis of by-products will contribute to enhancing 3-HP yield.

### 3.2 Blocking the synthesis of by-products improves 3-HP production

In our previous studies, it has been demonstrated that *Adh2A* and *Ach1* are key genes in *K*. *marxianus* responsible for mediating ethanol and acetate synthesis, respectively [17]. Therefore, based on YLJC01, strains with *Adh2A* knockout (YLJC03), *Ach1* knockout (YLJC04), and dual knockout of both *Adh2A* and *Ach1* (YLJC06) were constructed. Their abilities to synthesize 3-HP using YPD, YPF, and YPI were compared. As shown in Fig. 1B–D and Fig. S2, knocking out *Adh2A* significantly reduced ethanol production while increasing 3-HP yield by 0.48 %–6.24 %. Knocking out *Ach1* notably decreased acetate synthesis and enhanced 3-HP production by 5.98 %–14.01 %. Moreover, simultaneously knocking out both *Adh2A* and *Ach1* effectively blocked the synthesis of ethanol and acetate, ultimately improving 3-HP yield by 8.25 %–37.96 %. Among them, YLJC06 exhibited the best performance in synthesizing 3-HP using YPI, producing 2.49 g/L, 2.82 g/L, 2.98 g/L, and 2.62 g/L of 3-HP at 30 °C, 37 °C, 42 °C, and 45 °C respectively (Fig. 1B–D and Fig. S2). Therefore, the optimal temperature for 3-HP synthesis in strain YLJC06 was determined to be 42 °C.

### 3.3 Synthesis of 3-HP by YLJC01 using glycerol at 30 °C, 37 °C, and 42 °C

**Fig. 2:**
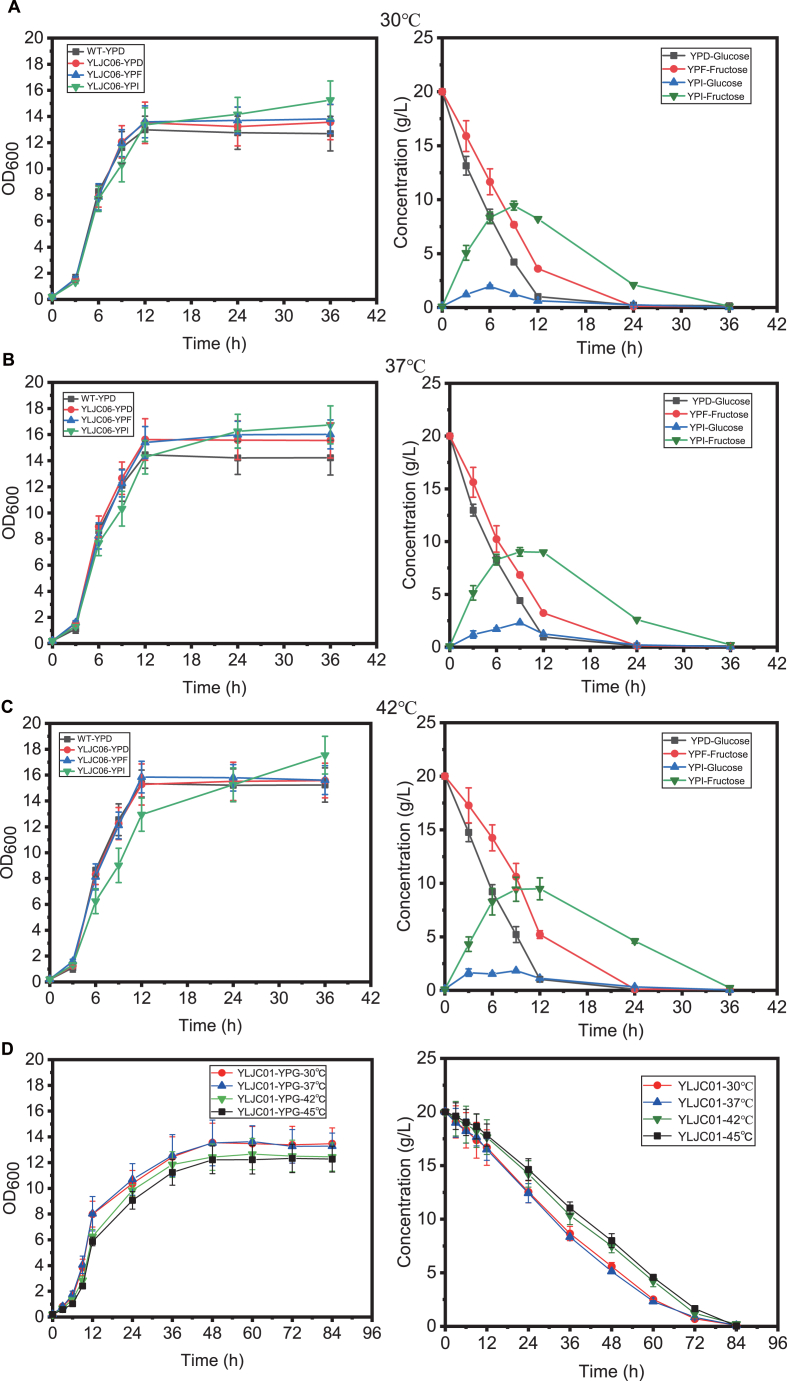
The growth curve and substrate consumption of strain YLJC06 utilizing YPD, YPF, and YPI for the synthesis of 3-HP at 30 °C (A), 37 °C (B), and 42 °C (C). (D) The growth curve and substrate consumption of strain YLJC01 utilizing YPG for the synthesis of 3-HP at 30 °C, 37 °C, 42 °C, and 45 °C. Data are shown as the mean ± SD from at least three experiments.

We further investigated the differences in 3-HP synthesis by YLJC01 using the non-fermentable carbon source glycerol at 30 °C, 37 °C, 42 °C, and 45 °C. As shown in Fig. 1E, YLJC01 produced 9.21 g/L, 10.25 g/L, 12.14 g/L, and 9.32 g/L of 3-HP from glycerol at 30 °C, 37 °C, 42 °C, and 45 °C, respectively. These yields were significantly higher than those achieved using glucose, fructose, or inulin. The significant difference in 3-HP production between fermentable carbon sources like glucose and the non-fermentable carbon source glycerol is attributed to our observation that yeast rapidly converts substantial substrates into by-products such as ethanol when utilizing fermentable carbon sources for fast growth, resulting in lower 3-HP yield, whereas its slower growth on glycerol allows sufficient time for MCR^N940V/K1106W/S1114R^ to convert the glycerol into 3-HP (Fig. 1B–E). To verify this hypothesis, we examined the growth curves and substrate consumption profiles of YLJC06 using YPD, YPF, and YPI, as well as of YLJC01 using YPG. As shown in Fig. 2A–C, when YLJC06 utilized inulin, compared to glucose and fructose, the additional hydrolysis step allowed for slower substrate utilization, thereby promoting higher 3-HP synthesis (Fig. 2A–C). In contrast, glycerol, as a non-fermentable carbon source, is consumed at a slower rate than fermentable carbon sources and does not generate by-products during its metabolism, making it more favorable for 3-HP accumulation (Fig. 2D).

From Fig. 1E, we also observed higher 3-HP yields at elevated temperatures, this occurs because enzymatic activity rises with increasing temperature, within a specific range. Moreover, since both the synthesis MSA and conversion of MSA to 3-HP requires NADPH as a coenzyme (Fig. 1A), and heat stress induces a stress response in the yeast, leading to increased NADPH production for combating reactive oxygen species (ROS) and other stress-related processes [23], which in turn enhances 3-HP synthesis. Therefore, we measured the intracellular NADPH levels in YLJC01 at different temperatures. As shown in Fig. 1E, the NADPH content indeed increased with rising temperature form 397.56 nmol/g DCW at 30 °C to 475.24 nmol/g DCW and 545.41 nmol/g DCW at 30 °C and 42 °C, suggesting that NADPH supply is a bottleneck for 3-HP synthesis.

### 3.4 Employing fermentation engineering strategies to enhance 3-HP production by YLJC06 using Jerusalem artichoke tubers powder

**Fig. 3:**
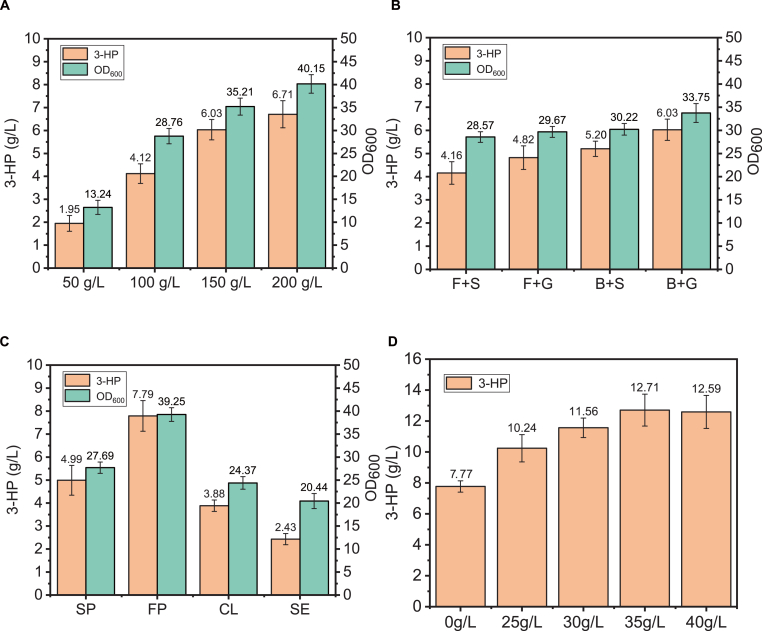
(A) YLJC06 synthesizes 3-HP using 50 g/L, 100 g/L, 150 g/L, and 200 g/L Jerusalem artichoke tuber powder. (B) YLJC06 synthesizes 3-HP using Jerusalem artichoke tuber powder with flasks sealed with sealing film (F + S), flasks wrapped with gauze (F + G), baffled flasks sealed with sealing film (B + S), and baffled flasks wrapped with gauze (B + G). (C) YLJC06 synthesizes 3-HP using Jerusalem artichoke tuber powder medium with soybean peptone (SP), fish meal peptone (FP), corn steep liquor (CL), and soybean meal extract (SE) as industrial nitrogen sources. (D) YLJC06 synthesizes 3-HP using 100 g/L Jerusalem artichoke tubers powder and 30 g/L fish meal peptone supplementing with 25 g/L, 30 g/L, 35 g/L, and 40 g/L CaCO_3_. Data are shown as the mean ± SD from at least three experiments.

For better alignment with industrial fermentation practices, Jerusalem artichoke tubers powder was utilized for 3-HP synthesis, with inulin content at 56 %. As shown in Fig. 3A, different concentrations (50 g/L, 100 g/L, 150 g/L and 200 g/L) of Jerusalem artichoke tubers powder as carbon source with YP nitrogen source at 37 °C yielded 1.95 g/L, 4.12 g/L, 6.03 g/L, and 6.71 g/L 3-HP with yields of 0.039, 0.041, 0.040, and 0.034 g/g respectively, indicating 100 g/L as optimal substrate loading since higher concentrations increased titer but decreased yield, causing carbon source wastage. To evaluate oxygen transfer effects, flasks sealed with film (F + S), gauze-wrapped flasks (F + G), baffled flasks sealed with film (B + S), and gauze-wrapped baffled flasks (B + G) containing YP medium with 100 g/L Jerusalem artichoke tubers powder produced 4.16 g/L, 4.82, g/L 5.20 g/L, and 6.03 g/L 3-HP respectively, demonstrating enhanced aeration promotes 3-HP synthesis while reducing ethanol production. Comparative assessment of four industrial nitrogen sources revealed fish meal peptone as optimal, elevating 3-HP production to 7.79 g/L. Additionally, calcium carbonate has been demonstrated to promote malonyl-CoA synthesis during the 3-HP production process. The key step in the biosynthesis of 3-HP via the malonyl-CoA pathway relies on the reaction catalyzed by acetyl-CoA carboxylase (ACC1) (Fig. 1A), which consumes bicarbonate ions (HCO_3_^−^) as a crucial substrate to carboxylate acetyl-CoA into malonyl-CoA. Meanwhile, the addition of calcium carbonate also helps mitigate the pH decrease caused by 3-HP synthesis (Qin et al., 2020). Therefore, the role of calcium carbonate in 3-HP synthesis was also investigated. As illustrated in Fig. 3D, consistent with reported CaCO_3_ benefits for 3-HP synthesis, supplementation at 25–40 g/L identified 35 g/L as optimal concentration, further boosting 3-HP titer to 12.71 g/L.

### 3.5 Strain YLJC06 produced 3-HP from Jerusalem artichoke tuber powder in a bioreactor

**Fig. 4:**
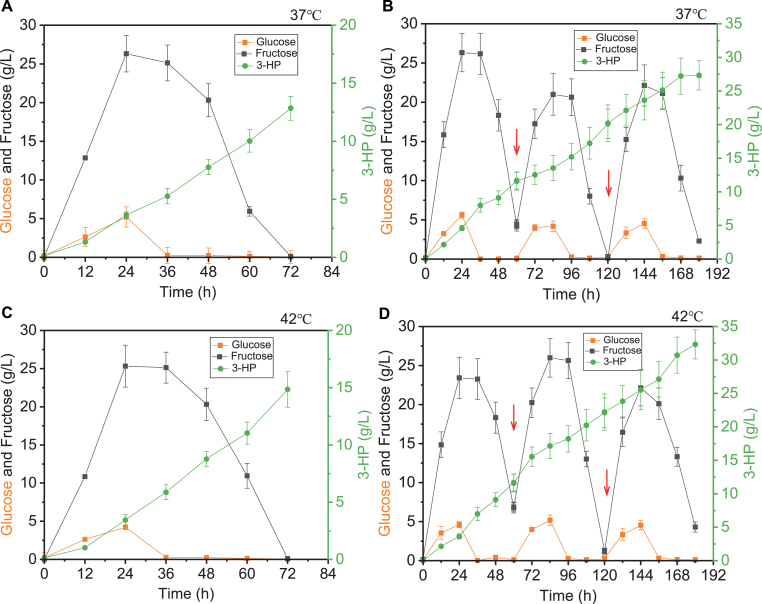
(A) YLJC06 synthesizes 3-HP using Jerusalem artichoke tuber powder medium in a fermenter at 37 °C with 35 g/L CaCO_3_. (B) YLJC06 synthesizes 3-HP using Jerusalem artichoke tuber powder medium in a fermenter at 42 °C with 35 g/L CaCO_3_. (C) YLJC06 synthesizes 3-HP through fed-batch fermentation using Jerusalem artichoke tuber powder medium in a fermenter at 42 °C with 35 g/L CaCO_3_. (D) YLJC06 synthesizes 3-HP through fed-batch fermentation using Jerusalem artichoke tuber at 42 °C. The arrows denote the feeding points. Data are shown as the mean ± SD from at least three experiments.

To better control oxygen and temperature conditions, strain YLJC06 was fermented in a bioreactor for 3-HP production. As shown in Fig. 4A and C, YLJC06 synthesized 12.84 g/L and 14.84 g/L of 3-HP using a medium containing 100 g/L Jerusalem artichoke tuber powder and fish meal peptone at 37 °C and 42 °C, respectively (Fig. 4A and C). Through two cycles of fed-batch fermentation, YLJC06 was able to synthesize 27.32 g/L and 32.31 g/L of 3-HP at 37 °C and 42 °C, respectively, using inulin as the substrate (Fig. 4B and D).

### 3.6 Reconstruction of the glycerol metabolic pathway in *K*. *marxianus*

**Fig. 5:**
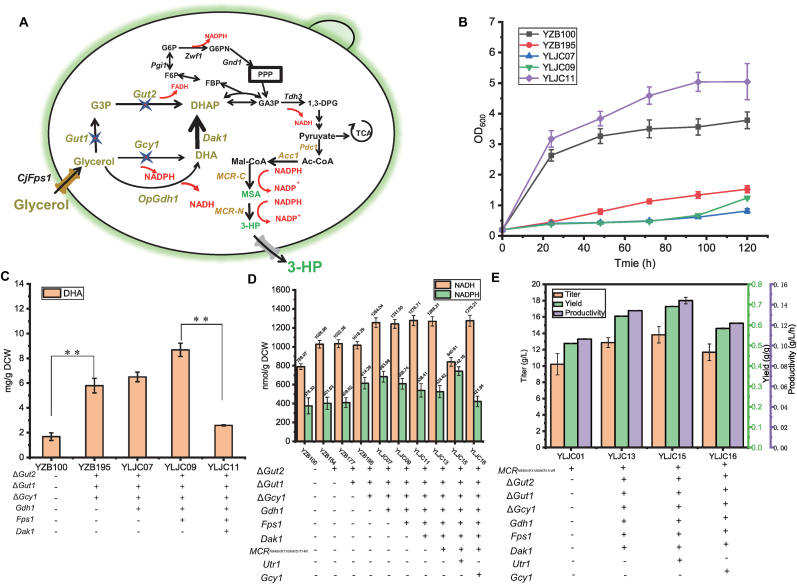
(A) The schematic diagram of the reconstructed glycerol metabolism pathway in *K. marxianus*. (B) The growth curves of strains YZB100, YZB195, YLJC07, YLJC09, and YLJC11 cultured with YNB medium supplemented with 2 % glycerol. (C) Comparison of the intracellular DHA concentrations in strains YZB100, YZB195, YLJC07, YLJC09, and YLJC11 cultured with YPG. (D) Comparison of intracellular NADH and NADPH concentrations in strains YZB100, YZB154, YZB177, YZB195, YLJC07, YLJC09, YLJC11, YLJC13, YLJC15, and YLJC16 cultured with YPG. (E) Comparison of 3-HP titer, yield and productivity of YLJC01, YLJC13, YLJC15 and YLJC16 cultured with YPG with 35 g/L CaCO_3_. Data are shown as the mean ± SD from at least three experiments. Significant differences with p < 0.05 are displayed with ∗ and significant differences with p < 0.01 are displayed with ∗∗.

To enhance the ability of *K*. *marxianus* to utilize glycerol for growth and compound synthesis, its glycerol metabolic pathway was reconstructed. Previous studies have elucidated that *K. marxianus* primarily utilizes glycerol through the GUT1/GUT2 pathway, generating the coenzyme FADH_2_ [15]. When this pathway is knocked out, the GCY1/DAK1 pathway can also be activated, endowing the yeast with a certain capacity for glycerol metabolism to produce the coenzyme NADPH, particularly in the presence of high concentrations of glycerol (Fig. 5A). The energy-generating efficiency of the coenzyme FADH_2_ is lower than that of NADH. Therefore, theoretically, pathways utilizing NADH as a coenzyme substrate have higher energy efficiency. Moreover, previous research has indicated that the high reducing power derived from glycerol utilization in *K. marxianus* originates from the NADH phosphorylation catalyzed by UTR1 [15]. Hence, we hypothesized that reconstructing an NAD^+^-dependent GDH1/DAK1 glycerol pathway could meet the energy demands for microbial growth while also addressing the NADPH requirements for certain synthetic reactions. Previous studies have demonstrated that replacing the native glycerol metabolic pathway in *S*. *cerevisiae* with the *Gdh1* from *O. parapolymorpha* and native Dak1 significantly enhances NADH supply, yielding engineered strains capable of ethanol production from glycerol [24]. Consequently, the *Gdh1* gene from *O. parapolymorpha* [24] was codon-optimized, synthesized, and transformed into a previously constructed triple-deficient *gut1gut2gcy1Δ* strain that had essentially lost its glycerol metabolic capability, resulting in the strain YLJC07. Surprisingly, YLJC07 exhibited weaker glycerol utilization ability compared to YZB195 (Fig. 5B). Unlike the typical yeast FPS1 that mediates glycerol efflux, FPS1 from *C. jadinii* facilitates glycerol uptake [24]. Therefore, the glycerol transporter *CjFps1* was codon-optimized, synthesized, and transformed into YLJC07 to obtain YLJC09; however, the transformation of *CjFps1* did not promote glycerol utilization in YLJC09 (Fig. 5B). It was speculated that the accumulation of DHA in the GDH1/DAK1 pathway inhibited the growth of the strain [25]. Therefore, the endogenous *Dak1* gene of *K. marxianus* was further recombinantly expressed, yielding the strain YLJC11. The research results demonstrated that the overexpression of *Dak1* significantly enhanced the glycerol utilization ability of YLJC11, which was 41.57 % higher than that of the wild-type yeast YZB100 (Fig. 5B).

To verify that the overexpression of *Dak1* restored the yeast's glycerol metabolic capability, the intracellular DHA content was measured. As shown in Fig. 5C, the results indicated that the intracellular DHA contents of YZB100, YZB195, YLJC07, YLJC09, and YLJC11 were 1.68 mg/g DCW 5.81 mg/g DCW, 6.50 mg/g DCW, 8.69 mg/g DCW, and 2.59 mg/g DCW, respectively. Overexpression of *Dak1* reduced the intracellular DHA content of YLJC011 by 70.19 %, bringing it to a level comparable to that of the wild type (Fig. 5C). To confirm that the GDH1/DAK1 pathway could provide more NADH and NADPH, the intracellular NADH and NADPH contents of YZB100, YZB154, YZB177, YZB195, YLJC07, YLJC09, YLJC11, and YLJC13 were measured. The results showed that the NADH content of YZB100, YZB154, YZB177, YZB195 were 789.97, 1026.96, 1032.35, and 1016.29 nmol/g DCW, respectively, while the NADH content of YLJC07, YLJC09, YLJC11, and YLJC13 were 1254.04, 1241.50, 1276.71, and 1288.21 nmol/g DCW, respectively. That is, the NADH levels after reconstruction of the glycerol metabolic pathway increased by approximately 20 % compared to pre-reconstruction. Similarly, NADPH increased by more than 10 % (Fig. 5D).

To demonstrate that the reconstruction of the glycerol metabolic pathway can promote the synthesis of 3-HP, the *MCR*^*N940V/K1106W/S1114R*^ gene was introduced into the yeast strain YLJC12, resulting in the strain YLJC13. As shown in Fig. 5E, the research findings indicate that when using YPG as the substrate, the 3-HP titer of YLJC01 is 10.21 g/L, whereas the 3-HP yield of YLJC13 is 12.87 g/L, which is 26.05 % higher than that of YLJC01 (Fig. 5E). Besides, the yield of YLJC01 and YLJC13 were 0.51 and 0.64 g/g, and the productivity were 0.11 and 0.13 g/L/h, respectively (Fig. 5E). This suggests that the yeast strain utilizing the GDH1/DAK1 pathway for glycerol metabolism not only exhibits stronger growth capability but also has a greater ability to synthesize compounds from glycerol, providing a reference for the synthesis of other glycerol-based compounds.

As previously demonstrated, NADPH supply is a bottleneck in 3-HP synthesis. Earlier studies have also indicated that the primary source of NADPH in *K. marxianus* when utilizing glycerol is the conversion of NADH to NADPH via UTR1. Therefore, we attempted to overexpress *Utr1* to enhance 3-HP synthesis, resulting in strain YLJC15. Additionally, theoretically, the GCY1/DAK1 pathway can also provide NADPH. However, when constructing the GDH1/DAK1 glycerol metabolic pathway, to ensure that glycerol utilization relied on the GDH1/DAK1 pathway, GCY1 was knocked out. To verify whether the GCY1/DAK1 pathway could enhance 3-HP synthesis by supplying NADPH, we complemented the expression of the *Gcy1* gene, yielding strain YLJC16. As shown in Fig. 5E, fermentation results indicated that YLJC15 synthesized 13.82 g/L 3-HP, while YLJC16 synthesized 11.68 g/L 3-HP (Fig. 5E). Measurements of NADH and NADPH levels showed that overexpression of *Utr1* indeed facilitated the conversion of NADH to NADPH (Fig. 5D). Compared to YLJC13, NADH content in YLJC15 decreased by 33.72 %, while NADPH content increased by 41.47 %, consistent with previous reports (Fig. 5D) [15]. Furthermore, overexpression of *Gcy1* failed to enhance 3-HP production, possibly because GCY1 is an alternative glycerol metabolizing enzyme that functions under microaerophilic conditions, and it has a lower affinity for glycerol compared to GDH1 [26]. Consequently, the primary route for glycerol metabolism remains the GDH1/DAK1 pathway in YLJC16 under aerobic conditions. This is supported by the NADH and NADPH measurements, which show that complementing *Gcy1* expression did not increase the NADPH content in strain YLJC16 (Fig. 5D).

### 3.7 *K. marxianus* YLJC15 synthesizes 3-HP using a fermenter

**Fig. 6:**
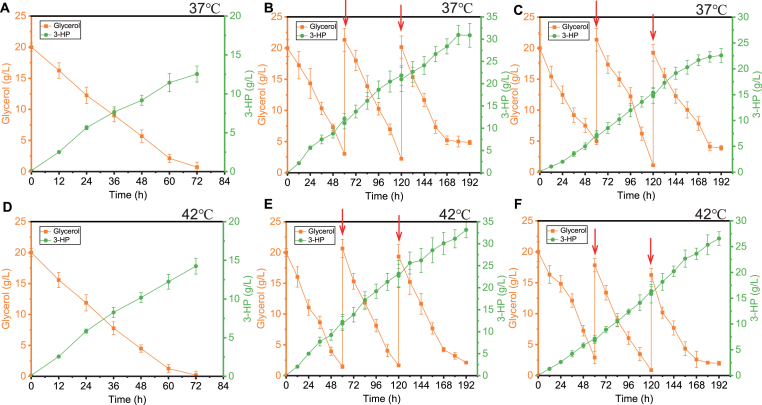
YLJC15 synthesizes 3-HP using YPG medium in a fermenter at 37 °C (A) and 42 °C (D). YLJC15 synthesizes 3-HP through fed-batch fermentation in a fermenter using glycerol at 37 °C (B) and 42 °C (E). YLJC15 synthesizes 3-HP through fed-batch fermentation using crude glycerol at 37 °C (C) and 42 °C (F). The arrows denote the feeding points. Data are shown as the mean ± SD from at least three experiments.

Fermenters offer more stable temperature control and a stronger oxygen supply compared to shake flasks. After being pre-cultured in YPD medium, YLJC15 was transferred to a 5 L fermenter (with a working volume of 2.5 L in YPG medium containing 35 g/L calcium carbonate) at an inoculation rate of 10 %. The agitation speed was set at 450 rpm, the temperature at 37 °C, and the aeration rate at 1 vvm. The research results indicate that YLJC15 synthesized 12.54 g/L of 3-HP in the fermenter, with a yield decreased 10.01 % (0.63 g/g) and productivity (0.17 g/L/h) increased 21.43 % comparable to that in shake flasks (Fig. 6A). Adequate fermenter aeration accelerated yeast growth and fermentation but lowered substrate conversion, mirroring our earlier results. Fermentation in the bioreactor at 42 °C yielded comparable results, with a 3-HP titer of 14.25 g/L, a yield of 0.71 g/g, and a productivity of 0.19 g/L/h (Fig. 6D). Subsequently, fed-batch fermentation was conducted. After two feedings at 37 °C, 30.95 g/L of 3-HP was synthesized. When a third feeding was attempted, the growth of the strain was inhibited (Fig. 6B). Under fed-batch fermentation conditions at 42 °C, YLJC15 was able to synthesize33.15 g/L of 3-HP, which was 7.11 % higher than that of 37 °C (Fig. 6E). To our knowledge, this is the first report of yeast-based 3-HP synthesis at temperatures above 40 °C. High-temperature fermentation not only reduces the risk of contamination during the fermentation process, enhances enzyme efficiency, but also effectively decreases the consumption of cooling water, offering significant energy-saving advantages.

During industrial fermentation processes, microorganisms generate significant amounts of heat. If the temperature is not controlled, the heat produced during microbial metabolism can lead to a rise in the fermentation system's temperature. Microbial growth and metabolism are highly sensitive to temperature, with an optimal temperature range being critical for efficient growth and metabolism. Both excessive and insufficient temperatures can negatively impact microbial activity, thereby affecting the yield and quality of fermentation products. Cooling water, through heat exchangers and other equipment, removes the heat generated during fermentation, maintaining the temperature within a suitable range. Traditional yeasts like *Saccharomyces cerevisiae* have an optimal growth and fermentation temperature of 30 °C, requiring substantial cooling water. In contrast, this project elevates the fermentation temperature of *K*. *marxianus* to 42 °C, an increase of 12 °C, significantly reducing cooling water consumption during industrial fermentation. Based on the formula governing the relationship between fermentation temperature and cooling water:

$$m=\frac{Q_{gen}-hA\left(T_{f}-T_{ambient}\right)}{c\cdot \Delta T_{water}}$$

Where: *m* = cooling water flow rate; *Q*_gen_ = heat generated during fermentation; *c* = specific heat capacity of water; *T*_*f*_ = fermentation temperature; *A* = heat dissipation area; *h* = natural heat dissipation coefficient; *T*_ambient_ = ambient temperature; Δ*T*_water_ = cooling water temperature difference.

Assuming a fermenter volume of 1000 tons (≈1000 m^3^, density ≈1000 kg/m^3^), fermentation heat generation *Q*_gen_ of 500 kW (a typical value, actual value depends on the process), ambient temperature *T*_ambient_ of 25 °C, cooling water inlet temperature *T*_in_ = 20 °C, outlet temperature *T*_out_ = 30 °C, temperature difference Δ*T*_water_ = 10 °C, heat transfer area *A* = 300 m^2^, initial fermentation temperature *T*_*f*1_ = 30 °C, elevated temperature *T*_*f*2_ = 42 °C, and annual operating time of 8000 h (≈333 days), substituting these values into the formula yields an annual cooling water saving of 155,000 tons, a 60 % reduction.

Converting this to carbon emission reductions: Based on an annual water saving of 155,000 tons, considering cooling water circulation requires pumps and refrigeration equipment, with a comprehensive energy consumption of 0.3 kWh/ton water (typical for industrial systems, ranging from 0.1 to 0.5 kWh/ton), and a power grid carbon emission factor of 0.581 kg CO_2_/kWh (2022 Chinese national average), the CO_2_ emission reduction can be calculated as:

CO_2_ emission reduction = water saving × energy consumption per ton of water × carbon emission factor.

Substituting the values: 155,000 tons × 0.3 kWh/ton × 0.581 kg CO_2_/kWh = 27,136.5 kg CO_2_ = 27.1 tons CO_2_/year. This means that for a single 1000-ton industrial fermenter, elevating the fermentation temperature alone can reduce CO_2_ emissions by 27.1 tons annually.

It is important to note that key parameters such as *Q*_*gen*_ (which depends on strain metabolism and product profile), *h* and *A* (which depend on fermenter design and scale), and system energy consumption can vary significantly. Therefore, this calculation serves primarily to demonstrate the potential magnitude of benefit from elevated fermentation temperatures, rather than to provide a precise forecast for any specific installation.

### 3.8 *K. marxianus* YLJC15 synthesizes 3-HP using crude glycerol

Industrial waste glycerol, also known as crude glycerol or glycerin, is a byproduct generated during various industrial processes, particularly in the production of biodiesel through transesterification of triglycerides (fats and oils) with methanol or ethanol. For every 100 kg of biodiesel produced, approximately 10 kg of crude glycerol is generated [11]. Additionally, crude glycerol can also originate from soap manufacturing, oleochemical production, and fatty acid esterification processes. Industrial crude glycerol typically contains a significant proportion of glycerol (around 60–85 %), along with various impurities such as methanol, water, salts, free fatty acids, soap residues, and catalysts used in the production process [27]. The disposal of industrial crude glycerol poses environmental challenges due to its high biological oxygen demand (BOD) and chemical oxygen demand (COD). Improper disposal can lead to water pollution and soil contamination [12]. Therefore, converting crude glycerol into the platform compound 3-HP not only contributes to reducing potential environmental pollution but also provides additional economic benefits. By utilizing the strain YLJC15 and substituting glycerol with crude glycerol as the fermentation substrate for fed-batch fermentation, the results, as depicted in Fig. 6C and F, show that at 37 °C, YLJC15 can synthesize 22.58 g/L of 3-HP using crude glycerol, and at 42 °C, it can synthesize 26.57 g/L of 3-HP (Fig. 6C and F).

## 4 Discussion

Currently, there are numerous research reports on cell factories utilizing *Escherichia coli*, *Klebsiella pneumoniae* or *Corynebacterium glutamicum* for the synthesis of 3-HP from glycerol or glucose (Table S2) [8,[28], [29], [30]]. Recombinant *E. coli* and *C. glutamicum* capable of synthesizing 3-HP from a combination of glucose and xylose have also been reported [27,31]. However, *K*. *pneumoniae* is a pathogenic bacterium and is not suitable for development into a cell engineering strain. Bacteria fermentation requires the additional supplementation of vitamin B12, which increases fermentation costs [6]. Moreover, bacteria often exhibit low acid tolerance, limiting the potential for higher-concentration product synthesis. Therefore, developing eukaryotic cell factories, such as yeast, represents a better choice [6]. In recent years, significant progress has been made in the research on the synthesis of 3-HP by yeast, with various yeast strains being engineered for the efficient production of this platform chemical with broad application prospects (Table S3) [6]. In the case of *S*. *cerevisiae*, researchers have achieved high-efficiency synthesis of 3-HP from glucose through rational metabolic engineering modifications, such as optimizing the expression of key enzyme genes and regulating energy metabolism. For instance, Yu *et*. *al.* utilized a super-engineered *S. cerevisiae* strain as a cellular platform and, through metabolic engineering strategies, achieved a high yield of 3-HP, reaching 4.4 g/L in batch fermentation and 56.5 g/L in fed-batch fermentation, which represents one of the highest reported yields of 3-HP produced by engineered microorganisms using glucose [1]. *Komagataella phaffii*, as a methylotrophic yeast capable of growing with methanol as the sole carbon and energy source, exhibits great potential in 3-HP synthesis. Àvila-Cabré *et*. *al.* has significantly improved the yield and productivity of 3-HP by *K*. *phaffii* through strategies such as optimizing metabolic pathways and introducing lactic acid transporters. For example, by overexpressing the lactic acid transporter Esbp6, the 3-HP concentration in a bioreactor reached 27.0 g/L, a 42 % increase compared to the parent strain [32]. Additionally, *Yarrowia lipolytica* is considered an ideal host for 3-HP production due to its exceptional tolerance to organic acids and lipid accumulation capabilities. Kang *et*. *al.* achieved a breakthrough in 3-HP production, reaching 100.37 g/L, by expanding the promoter toolkit of *Y. lipolytica* and employing a modular strategy to combine promoters with different characteristics to optimize the 3-HP synthesis pathway, setting a new benchmark for 3-HP synthesis in yeast systems [33]. In another study, Chen *et*. *al.* employed an engineered *Pichia pastoris* strain to produce 23 g/L of 3-HP using methanol through promoter engineering [34]. However, the yield of 3-HP synthesized from biomass resources is constrained by challenges such as yeast tolerance and substrate hydrolysis rates, resulting in relatively low production. For instance, Lertsriwong *et*. *al.* utilized engineered *S*. *cerevisiae* with rice straw hydrolysate as the substrate and achieved only 4.02 g/L of 3-HP [35]. These studies not only expand the technological means for 3-HP synthesis by yeast but also provide strong support for the industrial production of 3-HP. Compared to bacteria, *K*. *marxianus* exhibits stronger tolerance to osmotic pressure, acidity, and 3-HP toxicity, does not require the addition of vitamin B12 during fermentation, thus reducing fermentation costs, and is a food-safe microorganism. Compared to other yeasts, it has a broader range of substrate sources, stronger glycerol metabolism capabilities, higher growth and fermentation temperatures, and faster growth rates, although the titer of 3-HP produced by *K*. *marxianus* may be relatively lower than that achieved in other yeast platforms, potentially due to challenges like suboptimal precursor supply. These characteristics indicate that *K. marxianus* could be a potentially advantageous host over bacteria or other yeasts for developing a cellular factory to synthesize 3-HP from glycerol, holding promising development prospects.

## 5 Conclusions

This study successfully engineered *K. marxianus* to convert Jerusalem artichoke tuber powder and waste glycerol into 3-HP via the malonyl-CoA pathway. Using Jerusalem artichoke tuber powder, YLJC06 produced 32.31 g/L of 3-HP at 42 °C through fed-batch fermentation. Replacing the native glycerol pathway with an NADH-generating GDH1/DAK1 system enhanced glycerol utilization and cofactor supply. Coupled with heterologous expression of a high-activity *MCR* mutant and native *Utr1*, the strain YLJC15 achieved 33.15 g/L 3-HP at 42 °C using pure glycerol and 26.57 g/L with waste glycerol. Thermotolerant fermentation (42 °C) reduced cooling water needs by ∼60 % and lowered CO_2_ emissions by 27.1 tons/year per 1000-ton fermenter, demonstrating a viable, sustainable route for valorizing biodiesel waste into high-value biodegradable plastic precursors.

## CRediT authorship contribution statement

**Jiacheng Li:** Investigation. **Zhongmei Hu:** Investigation. **Yanjie Li:** Investigation. **Hao Zha:** Investigation. **Yujie Xie:** Investigation. **Mingtao Zhao:** Writing – review & editing, Formal analysis. **Lili Ren:** Writing – review & editing, Writing – original draft, Funding acquisition. **Biao Zhang:** Writing – review & editing, Writing – original draft, Conceptualization.

## Declaration of competing interest

The authors declare that they have no known competing financial interests or personal relationships that could have appeared to influence the work reported in this paper.
