# Supplementary material for: Engineering Kluyveromyces marxianus for 3-hydroxypropionic acid production at elevated temperature from Jerusalem artichoke tubers and crude glycerol
Source: Synth Syst Biotechnol. 2026 Jan 20;13:1–13. doi: 10.1016/j.synbio.2026.01.008 (PMC12856427; doi:10.1016/j.synbio.2026.01.008)
Supplement: Multimedia component 1 [file mmc1.doc]

**Engineering *Kluyveromyces marxianus* for 3‑hydroxypropionic acid production at elevated temperature from Jerusalem artichoke tubers and crude glycerol**

Jiacheng Li^b1^, Zhongmei Hu^b1^, Yanjie Li^b1^, Hao Zha^b^, Yujie Xie^b^, Mingtao Zhao^b^, Lili Ren^a,b*^, and Biao Zhang^a,b**^

^a^Key Laboratory of Green and Precise Synthetic Chemistry and Applications, Ministry of Education, Huaibei Normal University, Huaibei, Anhui 235000, P. R. China

^b^Anhui Province Key Laboratory of Pollutant Sensitive Materials and Environmental Remediation, School of Life Sciences, Huaibei Normal University, Huaibei, Anhui 235000, P. R. China

^*^Correspondence:

Lili Ren, renlili@chnu.edu.cn, Phone: +86 561-3802235, Fax: +86 561-3802235;

^**^Corresponding author:

Biao Zhang, zhangbiao@chnu.edu.cn, Phone: +86 561-3802235, Fax: +86 561-3802235.

^1^These authors contributed equally to this work.

**Table S1 Primers used in this study***

| Primers | Sequences |
| --- | --- |
| SNR52-SMAI-F | 5′-TCCCCCGGGTCTTTGAAAAGATAATGTATG-3′ |
| URA3-SNR52-R | 5′-CAACTAATCTTAACAACTCTGATCATTTATCTTTCACTGC-3′ |
| URA3-CYC1T-F | 5′-AGAGTTGTTAAGATTAGTTGGTTTTAGAGCTAGAAATAGC-3′ |
| CYC1T-SMAI-R | 5′-TCCCCCGGGCAAATTAAAGCCTTCGAGC-3′ |
| M13-F | 5′-CGCCAGGGTTTTCCCAGTCACGAC-3′ |
| M13-R | 5′-AGCGGATAACAATTTCACACAGGA-3′ |
| XYL2-M13-F | 5′-ATGACCAACACTCAAAAAGCCGTTGTTTTGAAGAAGCAAGGAGAGATTGCTTTCGAAGAGAGGGTTTTCCCAGTCACGAC-3′ |
| XYL2-M13-R | 5′-TCATTCTGGACCATCAATGATAGTCTTGACAACTTCATTACCGTGATCTCTGTTGAAATTAGCGGATAACAATTTCACAC-3′ |
| XYL2-SNR52-R | 5′-GTCTTCTTGATGTGAACCTTGATCATTTATCTTTCACTGC-3′ |
| XYL2-CYC1T-F | 5′-AAGGTTCACATCAAGAAGACGTTTTAGAGCTAGAAATAGC-3′ |
| XYL1-M13-F | 5′-ATGACATACCTCGCACCAACAGTTACCTTGAACAATGGATCCAAGATGCCAGGGTTTTCCCAGTCACGAC-3′ |
| M13UPRM13-F | 5′-GCATGCAAGCTTGGCGTAATCATGGTCATAGCTGTTTCCTGTGTGAAATTGTTATCCGCTTGCAAGGCGATTAAGTTGGG-3′ |
| XYL1-M13-R | 5′-TTAGATAAAGGTTGGGAATTCGTTGCCCAACCAGTCCCATGGGTCGTTAAAGCGGATAACAATTTCACAC-3′ |
| XYL1-SNR52-R | 5′-CGTCTTGTTGAAGTGGTCCAGATCATTTATCTTTCACTGC-3′ |
| XYL1-CYC1T-F | 5′-TGGACCACTTCAACAAGACGGTTTTAGAGCTAGAAATAGC-3′ |
| LAC4-M13-F1 | 5′-ATGTCTTGCCTTATTCCTGAGAATTTAAGGAACCCTAAAAAGGTTCACGAAAATAGATTGAGGGTTTTCCCAGTCACGAC-3′ |
| LAC4-M13-R1 | 5′-CCATTCTCCTCGTTCAACAAAGAGCTTGCATCGTAAACTTTAGATCCATCTTCAGGTTCGAGCGGATAACAATTTCACAC-3′ |
| LAC4-SNR52-R1 | 5′-GAGAGCCACCATTGATCTTGGATCATTTATCTTTCACTGC-3′ |
| LAC4-SNR52-R1 | 5′-CAAGATCAATGGTGGCTCTCGTTTTAGAGCTAGAAATAGC-3′ |
| KmURA3-F | 5′-ATGTCGACTAAGAGTTACTCGGAA-3′ |
| KmURA3-R | 5′-TTAAGCGGATCTGCCTACT-3′ |
| URA3-F | 5′-ATGTCGACTAAGAGTTACTC-3′ |
| URA3-R | 5′-TTAAGCGGATCTGCCTACTC-3′ |
| XYL2-F | 5′-ATGACCAACACTCAAAAAGCCGT-3′ |
| XYL2-R | 5′-TCATTCTGGACCATCAATGATAGT-3′ |
| XYL1-F | 5′-ATGACATACCTCGCACCAAC-3′ |
| XYL1-R | 5′-TTAGATAAAGGTTGGGAATTC-3′ |
| LAC4-F1 | 5′-ATGTCTTGCCTTATTCCTGAG-3′ |
| LAC4-R1 | 5′-CCATTCTCCTCGTTCAACAAAG-3′ |
| LAC4-M13-F2 | 5′-TCCAGGGTTCTTCTTATGATCACATCAATTTCACACTTTACG  AACCTGAAGATGGATCTAAGGGTTTTCCCAGTCACGAC-3′ |
| LAC4-M13-R2 | 5′-CTTAGGACAAGTTGGGAAGCTCTGTCTAAGTACGCGACCT  CGTACTCTGGATTATCTGATAGCGGATAACAATTTCACAC-3′ |
| LAC4-SNR52-R2 | 5′-CTTGTCTGAAACCAACATGGGATCATTTATCTTTCACTGC-3′ |
| LAC4-SNR52-R2 | 5′-CCATGTTGGTTTCAGACAAGGTTTTAGAGCTAGAAATAGC-3′ |
| LAC4-F2 | 5′-TCCAGGGTTCTTCTTATGATC3′ |
| LAC4-R2 | 5′-CTTAGGACAAGTTGGGAAGC-3′ |
| ADH2A-F | 5'-ATGTCTATTCCAACTACTCAAAAG-3' |
| ADH2A-R | 5'-TTATTTGGAAGTGTCAACAACGTATC-3' |
| ADH2A-M13F-F | 5'-ATGTCTATTCCAACTACTCAAAAGGGTGTTATCTTCTACGAAAACGGTGGTCAATTGTACAGGGTTTTCCCAGTCACGAC-3' |
| ADH2A-M13R-R | 5'-TTATTTGGAAGTGTCAACAACGTATCTACCTAGAATCTTACCTTGTTCCATCTTTTCGTAAGCGGATAACAATTTCACAC-3' |
| CR-ADH2A-SNR52-R | 5'-TACCGTCGTTCTAGTCGGTTTGCATCTACAACAGTAGAAATTATTTAAAGTTC-3' |
| CR-ADH2A-CYC1T-F | 5'-GCAAACCGACTAGAACGACGGTAAATTTCTACTGTTGTAGATCGTCTCTG-3' |
| ACH1-M13-F | 5'-ATGACAGTTTCTAGATTGTTGAAAGAGAGAGTGAGATATGCGCCATATTTGAAGAAGGTGAGGGTTTTCCCAGTCACGAC-3' |
| ACH1-M13-R | 5'-GTATGGTGGGTTCACTGGCATATCGATGTCGTGCAAACCTTCGAACGAAGGGGTTGCTGTAGCGGATAACAATTTCACAC-3' |
| CR-ACH1-SNR52-R | 5'-TCACAATTTGGTGGAATTCTTCGATCTACAACAGTAGAAATTATTTAAAGTTC-3' |
| CR-ACH2-CYC1T-F | 5'-CGAAGAATTCCACCAAATTGTGAAATTTCTACTGTTGTAGATCGTCTCTG-3' |
| ACH1-F | 5'-ATGACAGTTTCTAGATTGTTG-3' |
| ACH1-R | 5'-GTATGGTGGGTTCACTGGCAT-3' |

* The sgRNA sequences are indicated shadowed, the restricted enzyme sites are indicated underlined.

**Table S2 3-HP-producing bacterial strains**

| **Microorganisms** | **Carbon Source** | **Pathway** | **Temperature** | **Titer (g/L)** | **Yield（g/g）** | **Productivity (g/(L·h))** | **References** |
| --- | --- | --- | --- | --- | --- | --- | --- |
| *K. pneumoniae* | Glycerol | Glycerol oxidation via the coenzyme A-independent pathway | 37 ℃ | 102.6 | 0.86 | 1.07 |  |
| *K. pneumoniae* | Glycerol | Glycerol oxidation via the coenzyme A-independent pathway | 37 ℃ | 88.8 |  | 0.98 |  |
| *K. pneumoniae* | Glycerol | Glycerol oxidation via the coenzyme A-independent pathway | 37 ℃ | 83.8 | 0.59 | 1.16 |  |
| *E. coli* | Glycerol | Glycerol oxidation via the coenzyme A-independent pathway | 37 ℃ | 76.2/61 | 0.45/0.594 | 1.89/2.28 |  |
| *E. coli* | Glucose and glycerol | Glycerol oxidation via the coenzyme A-independent pathway | 37 ℃ | 71.9 |  | 1.8 |  |
| *E. coli* | Glycerol | Glycerol oxidation via the coenzyme A-independent pathway | 37 ℃ | 63.05 |  |  |  |
| *E. coli* | Glucose | Malonyl-CoA pathway | 37 ℃ | 49.04 |  | 0.71 |  |
| *E. coli* | Acetate | Malonyl-CoA pathway | 37 ℃ | 15.8 | 0.71 | 0.39 |  |
| *E. coli* | Ethanol | Malonyl-CoA pathway | 37 ℃ | 13.1 | 0.57 | 0.14 |  |
| *E. coli* | Fatty acids | Malonyl-CoA pathway | 37 ℃ | 52 | 1.56 | 1.13 |  |
| *E. coli* | Malonate | Malonyl-CoA pathway | 37 ℃ | 1.2 |  |  |  |
| *E. coli* | Glucose | β-alanine pathway | 37 ℃ | 31.1 | 0.423 | 0.63 |  |
| *E. coli* | Glucose and glycerol | dhaB、dhaR | 37 ℃ | 14.3 | 0.14 | 0.26 |  |
| *L. reuteri*and *E. coli* | Glycerol | Glycerol oxidation via the coenzyme A-dependen | 37 ℃ | 125.93 | 0.95 | 2.47 |  |
| *C. glutamicum* | Glucose or (Glucose and xylose) | Glycerol oxidation via the coenzyme A-independent pathway | 30 ℃ | 62.6/54.8 | 0.51/0.49 | 0.86/0.76 |  |
| *C. glutamicum* | Glucose | β-alanine pathway | 37℃ | 47.54 | 0.295 | 0.475 |  |
| *C. glutamicum* | Acetate | Malonyl-CoA pathway | 37 ℃ | 17.1 | 0.1 | 0.14 |  |
| *H.bluephagenesis* | 1,3-Propanediol | 1,3-Propanediol pathway | 37 ℃ | 154 | 0.93 | 2.4 |  |
| *G. oxydans* | Glucose and 1,3-Propanediol | 1,4-Propanediol pathway | 30 ℃ | 45.8 | 1.14 | 1.86 |  |
| *B. subtilis* | Glycerol | Glycerol oxidation via the coenzyme A-independent pathway | 37 ℃ | 10 | 0.79 |  |  |
| *P.denitrificans* | Glycerol or Crude glycerol | Glycerol oxidation via the coenzyme A-independent pathway | 30 ℃ | 102/65 |  | 2.5/ |  |
| *P.asiatica C1* | Glucose and glycerol | Glycerol oxidation via the coenzyme A-independent pathway | 30 ℃ | 63 | 0.96 | 2.15 |  |
| [*S.elongatus PCC 7942.*](https://www.sciencedirect.com/topics/agricultural-and-biological-sciences/synechococcus-elongatus) | CO_2_ | Malonyl-CoA pathway | 37 ℃ | 0.83 |  |  |  |
| *M.trichosporium OB3b* | CH_4_ | Malonyl-CoA pathway | 30 ℃ | 0.6 | 0.02 |  |  |
| *Bacillus cereus* | Glucose | β-alanine pathway | 30 ℃ | 13.77 | 0.147 | 0.172 |  |
| *K. pneumoniae* and *G. oxydans* | Glycerol | Glycerol oxidation via the coenzyme A-independent pathway | 30 ℃/37 ℃ | 60.5 | 0.5 | 1.12 |  |

**Table S3 3-HP-producing yeast strains**

| **Microorganisms** | **Carbon Source** | **Pathway** | **Temperature** | **Titer (g/L)** | **Yield（g/g）** | **Productivity (g/(L·h))** | **References** |
| --- | --- | --- | --- | --- | --- | --- | --- |
| *P. pastoris* | Glycerol | Malonyl-CoA pathway | 30 ℃ | 24.75 | 0.13 | 0.54 |  |
| *P. pastoris* | Glycerol | Malonyl-CoA pathway | 30 ℃ | 37.05 | 0.18 | 0.71 |  |
| *P. pastoris* | Methanol | Malonyl-CoA pathway | 30 ℃ | 48.2 | 0.23 | 0.204 |  |
| *P. pastoris* | Methanol | β-alanine pathway | 28℃ | 27 | 0.19 | 0.56 |  |
| *S. cerevisiae* | Glucose | Malonyl-CoA pathway | 30 ℃ | 9.8 | 0.06 | 0.09 |  |
| *S. cerevisiae* | Glucose | β-alanine pathway | 30 ℃ | 25 | 0.25 |  |  |
| *S. cerevisiae* | Glucose | Oxaloacetate pathway | 30 ℃ | 18.1 | 0.125 | 0.17 |  |
| *S. cerevisiae* | Glucose and CaCO3 | Malonyl-CoA pathway | 30 ℃ | 11.25 |  | 0.156 |  |
| *S. cerevisiae* | Glucose | Malonyl-CoA pathway | 30 ℃ | 56.5 | 0.31 |  |  |
| *Y. lipolytica* | Glucose | Malonyl-CoA pathway | 30 ℃ | 16.23 | 0.054 | 0.067 |  |
| *Y.lipolytica* | Glucose | Malonyl-CoA pathway |  | 100.37 | 0.21 | 0.48 |  |
| *S. pombe* | Cellobiose | Malonyl-CoA pathway | 30 ℃ | 11.4 | 0.11 | 0.14 |  |
| *D. hansenii* | Glucose and propionic acid |  | 30 ℃ | 62.42 |  | 1.3 |  |
| *K. marxianus* | JAT and CaCO_3_ | Malonyl-CoA pathway | 37 ℃ | 27.32 | / | 0.14 | This study |
| *K. marxianus* | JAT and CaCO_3_ | Malonyl-CoA pathway | 42 ℃ | 32.31 | / | 0.17 | This study |
| *K. marxianus* | Glycerol and CaCO_3_ | Malonyl-CoA pathway | 37 ℃ | 30.95 | 0.52 | 0.16 | This study |
| *K. marxianus* | Glycerol and CaCO_3_ | Malonyl-CoA pathway | 42 ℃ | 33.15 | 0.55 | 0.17 | This study |
| *K. marxianus* | Waste glycerol and CaCO_3_ | Malonyl-CoA pathway | 37 ℃ | 22.58 | 0.38 | 0.12 | This study |
| *K.marxianus* | Waste glycerol and CaCO_3_ | Malonyl-CoA pathway | 42 ℃ | 26.57 | 0.44 | 0.14 | This study |

**Table S4 The gene sequences used in this study**

| *OpGdh1* | ATGAAGGGTTTGTTGTACTACGGTACTAACGACATCAGATACTCTGAAACTGTTCCAGAACCAGAAATCAAGAACCCAAACGACGTTAAGATCAAGGTTTCTTACTGTGGTATCTGTGGTACCGATTTGAAGGAATTCACTTACTCTGGTGGTCCAGTTTTCTTCCCAAAGCAAGGTACCAAGGATAAGATCTCTGGTTACGAATTGCCATTGTGTCCAGGTCACGAATTCTCTGGTACCGTTGTTGAAGTTGGTTCCGGTGTTACTTCTGTTAAGCCAGGTGACAGAGTTGCTGTTGAAGCTACTTCTCACTGTTCTGACAGATCTAGATACAAGGACACTGTTGCTCAAGATTTGGGTTTGTGTATGGCTTGTCAATCCGGTTCTCCAAACTGTTGTGCTTCTTTGTCTTTCTGTGGTTTGGGTGGTGCTTCCGGTGGTTTCGCTGAATACGTTGTTTACGGTGAAGACCACATGGTTAAGTTGCCAGACTCTATCCCAGACGATATCGGTGCTTTGGTTGAACCAATCTCCGTTGCTTGGCACGCTGTTGAAAGAGCTAGATTCCAACCAGGTCAAACCGCTTTGGTTTTGGGTGGTGGTCCAATCGGTTTGGCTACCATCTTGGCTTTGCAAGGTCACCACGCTGGTAAAATCGTTTGTTCCGAACCAGCTTTGATCAGAAGACAATTCGCTAAGGAATTGGGTGCTGAAGTTTTCGATCCATCCACCTGTGACGATGCTAACGCTGTTTTGAAGGCTATGGTTCCAGAAAACGAAGGTTTCCACGCTGCTTTCGACTGTTCCGGTGTTCCACAAACCTTCACCACCTCCATCGTTGCTACCGGTCCATCTGGTATCGCTGTTAACGTTGCTGTTTGGGGTGACCACCCAATCGGTTTCATGCCAATGTCTTTGACTTACCAAGAAAAGTACGCTACTGGTTCTATGTGTTACACCGTTAAGGATTTCCAAGAAGTTGTTAAGGCTTTGGAAGACGGTTTGATCTCTTTGGACAAGGCTAGAAAGATGATCACCGGTAAAGTTCACTTGAAGGACGGTGTTGAAAAGGGTTTCAAGCAATTGATCGAACACAAGGAAAACAACGTTAAGATCTTGGTTACCCCAAACGAAGTTTCTTAA |
| --- | --- |
| *CjPfs1* | ATGACTGGTGAATTGTTGGCTTCTGGTGAAGGTTGTTCCTCCGACATCGTTTTGACCAACTCTACTGCTGCTCCATCCTCCGGTTTGGAAAGAAGAGCTAACATCACTGATCACATCTCTTGTGAACACTTCACTGCTTTGCAAAGATTCAGATACGGTTTCAGAGAATACTTCGCTGAATTCATCGGTACCATGATCTTGGTTATGTTCGGTGACGGTGTTGTTGCTCAATACACTTTGTCCAAGGGTTCTGCTGGTAACTACACCACCATCGCTTTCTCCTGGGCTACCGCTGTTTTCTTGGGTTACTGTTGTTCCGCTGGTATCTCTGGTGCTCACTTGAACCCAGCTGTTACCTTGTCTGCTGCTACCTTCAGACAATTCCCATGGAGAAAGGTTTTGGGTTACATGTTCGCTCAAGGTTTGGGTGGTTACATCGGTGCTTTGATCGTTTACGGTACCTACATCCAATCTATCAACAACTACTCCGGTGAAGGTCAAAGAATCGCTGTTGGTGACAAGTCCACCGGTGGTATCTTCTGTACTTTCCCACAACCATACTTGAACACCAAGGGTCAAGTTACCTCCGAATTGGTTACCACCGCTTTGTTGCAATTCGGTATCTTCTCCATGACCGATCCACACAACGCTCCATTGGGTAACTTCTTCCCATTCGGTTTGTGGATCTTGATCTACGGTTTGGGTACCTCTTTCGGTTACCAAACCGGTTACGCTATCAACTTCGCTAGAGATTTCACCCCAAGATTGGCTGCTTTGACCGTTGGTTACGGTACCGAAATGTTCACTGCTTACTACCACTACTTCTGGGTTCCAATGATCATCCCATTCATCGGTGCTTTGTTGGGTGCTTTCATCTACGATTTCTTCATCTACCAAGGTTTGGATTCCCCATTGAACCAACCAAAGTTCGGTTACGACATCAGAAAGAAGAAGATCCAAGAATTCGAATTCAAGTTGGAAAACTACAAGTTGGATTTCAACCCAGAAGCTTCCCACGGTAGATTGGACGCTTAA |
| *CaMCR*^N940V/K1106W/S1114R10^ | ATGTCTGGTACTGGTAGATTGGCTGGTAAAATCGCTTTGATCACTGGTGGTGCTGGTAACATCGGTTCTGAATTGACTAGAAGATTCTTGGCTGAAGGTGCTACCGTTATCATCTCTGGTAGAAACAGAGCTAAGTTGACTGCTTTGGCTGAAAGAATGCAAGCTGAAGCTGGTGTTCCAGCTAAGAGAATCGATTTGGAAGTTATGGATGGTTCTGACCCAGTTGCTGTTAGAGCTGGTATCGAAGCTATCGTTGCTAGACACGGTCAAATCGACATCTTGGTTAACAACGCTGGTTCTGCTGGTGCTCAAAGAAGATTGGCTGAAATCCCATTGACTGAAGCTGAATTGGGTCCAGGTGCTGAAGAAACCTTGCACGCTTCTATCGCTAACTTGTTGGGTATGGGTTGGCACTTGATGAGAATCGCTGCTCCACACATGCCAGTTGGTTCTGCTGTTATCAACGTTTCCACCATCTTCTCCAGAGCTGAATACTACGGTAGAATCCCATACGTTACCCCAAAGGCTGCTTTGAACGCTTTGTCCCAATTGGCTGCTAGAGAATTGGGTGCTAGAGGTATCAGAGTTAACACTATCTTCCCAGGTCCAATCGAATCTGATAGAATCAGAACTGTTTTCCAAAGAATGGACCAATTGAAGGGTAGACCAGAAGGTGACACCGCTCACCACTTCTTGAACACTATGAGATTGTGTAGAGCTAACGACCAAGGTGCTTTGGAAAGAAGATTCCCATCTGTTGGTGACGTTGCTGACGCTGCTGTTTTCTTGGCTTCTGCTGAATCCGCTGCTTTGTCTGGTGAAACCATCGAAGTTACCCACGGTATGGAATTGCCAGCTTGTTCCGAAACCTCTTTGTTGGCTAGAACCGATTTGAGAACTATCGATGCTTCCGGTAGAACCACTTTGATCTGTGCTGGTGACCAAATCGAAGAAGTTATGGCTTTGACCGGTATGTTGAGAACTTGTGGTTCCGAAGTTATCATCGGTTTCAGATCCGCTGCTGCTTTGGCTCAATTCGAACAAGCTGTTAACGAATCCAGAAGATTGGCCGGTGCTGACTTCACCCCACCAATCGCTTTGCCATTGGACCCAAGAGATCCAGCTACCATCGACGCTGTTTTCGATTGGGCTGGTGAAAACACTGGTGGTATCCACGCTGCTGTTATCTTGCCAGCTACTTCCCACGAACCAGCTCCATGTGTTATCGAAGTTGATGACGAAAGAGTTTTGAACTTCTTGGCTGATGAAATCACCGGTACTATCGTTATCGCTTCTAGATTGGCTAGATACTGGCAATCTCAAAGATTGACTCCAGGTGCTAGAGCTAGAGGTCCAAGAGTTATCTTCTTGTCTAACGGTGCTGATCAAAACGGTAACGTTTACGGTAGAATCCAATCTGCTGCTATCGGTCAATTGATCAGAGTTTGGAGACACGAAGCTGAATTGGATTACCAAAGAGCTTCTGCTGCTGGTGACCACGTTTTGCCACCAGTTTGGGCTAACCAAATCGTTAGATTCGCTAACAGATCTTTGGAAGGTTTGGAATTTGCTTGTGCTTGGACTGCTCAATTGTTGCACTCTCAAAGACACATCAACGAAATCACCTTGAACATCCCAGCTAACATCTCTGCTACTACCGGTGCTAGATCTGCTTCTGTTGGTTGGGCTGAATCTTTGATCGGTTTGCACTTGGGTAAAGTTGCTTTGATCACCGGTGGTTCCGCTGGTATCGGTGGTCAAATCGGTAGATTGTTGGCTTTGTCCGGTGCTAGAGTTATGTTGGCTGCTAGAGACAGACACAAGTTGGAACAAATGCAAGCTATGATCCAATCTGAATTGGCTGAAGTTGGTTACACTGATGTTGAAGACAGAGTTCACATCGCTCCAGGTTGTGACGTTTCTTCCGAAGCTCAATTGGCTGATTTGGTTGAAAGAACTTTGTCTGCTTTCGGTACTGTTGACTACTTGATCAACAACGCTGGTATCGCTGGTGTTGAAGAAATGGTTATCGATATGCCAGTTGAAGGTTGGAGACACACTTTGTTCGCTAACTTGATCTCCAACTACTCTTTGATGAGAAAGTTGGCTCCATTGATGAAGAAGCAAGGTTCCGGTTACATCTTGAACGTTTCTTCTTACTTCGGTGGTGAAAAGGACGCTGCTATCCCATACCCAAACAGAGCTGACTACGCTGTTTCTAAGGCTGGTCAAAGAGCTATGGCTGAAGTTTTCGCTAGATTCTTGGGTCCAGAAATCCAAATCAACGCTATCGCTCCAGGTCCAGTTGAAGGTGACAGATTGAGAGGTACTGGTGAAAGACCAGGTTTGTTCGCTAGAAGAGCTAGATTGATCTTGGAAAACAAGAGATTGAACGAATTGCACGCTGCTTTGATCGCTGCTGCTAGAACTGACGAAAGATCCATGCACGAATTGGTTGAATTGTTGTTGCCAAACGATGTTGCTGCTTTGGAACAAAACCCAGCTGCTCCAACTGCTTTGAGAGAATTGGCTAGAAGATTCAGATCTGAAGGTGACCCAGCTGCTTCTTCCTCTTCTGCTTTGTTGAACAGATCCATCGCTGCTAAGTTGTTGGCTAGATTGCACAACGGTGGTTACGTTTTGCCAGCTGATATCTTCGCTAACTTGCCAAACCCACCAGACCCATTCTTCACCAGAGCTCAAATCGATAGAGAAGCTAGAAAGGTTAGAGATGGTATCATGGGTATGTTGTACTTGCAAAGAATGCCAACCGAATTTGATGTTGCTATGGCTACCGTTTACTACTTGGCTGATAGAGTTGTTTCTGGTGAAACTTTCCACCCATCCGGTGGTTTGAGATACGAAAGAACTCCAACCGGTGGTGAATTGTTCGGTTTGCCATCCCCAGAAAGATTGGCTGAGTTGGTTGGTTCCACCGTTTACTTGATCGGTGAACACTTGACCGAACACTTGAACTTGTTGGCTAGAGCTTACTTGGAAAGATACGGTGCTAGACAAGTTGTTATGATCGTTGAAACCGAAACCGGTGCTGAAACCATGAGAAGATTGTTGCACGATCACGTTGAAGCTGGTAGATTGATGACCATCGTTGCTGGTGACCAGATCGAAGCTGCTATCGATCAAGCTATCACCAGATACGGTAGACCAGGTCCAGTCGTTTGTACTCCATTCAGACCATTGCCAACCGTTCCATTGGTTGGTAGAAAGGATTCCGACTGGTCTACTGTTTTGTCCGAAGCTGAATTTGCTGAATTGTGTGAACACCAATTGACTCACCACTTCAGAGTTGCTAGATGGATCGCTTTGTCCGATGGTGCTAGATTGGCTTTGGTTACCCCAGAAACTACTGCTACTTCCACCACTGAACAATTCGCTTTGGCTAACTTCATCAAGACCACCTTGCACGCCTTCACTGCTACTATCGGTGTTGAATCTGAAAGAACTGCTCAAAGAATCTTGATCAACCAAGTTGACTTGACCAGAAGAGCTAGAGCTGAAGAACCAAGAGACCCACACGAAAGACAACAAGAATTGGAAAGATTCATCGAAGCTGTTTTGTTGGTTACTGCTCCATTGCCACCAGAAGCTGACACCAGATACGCTGGTAGAATCCACAGAGGTAGAGCTATCACTGTTTAA |


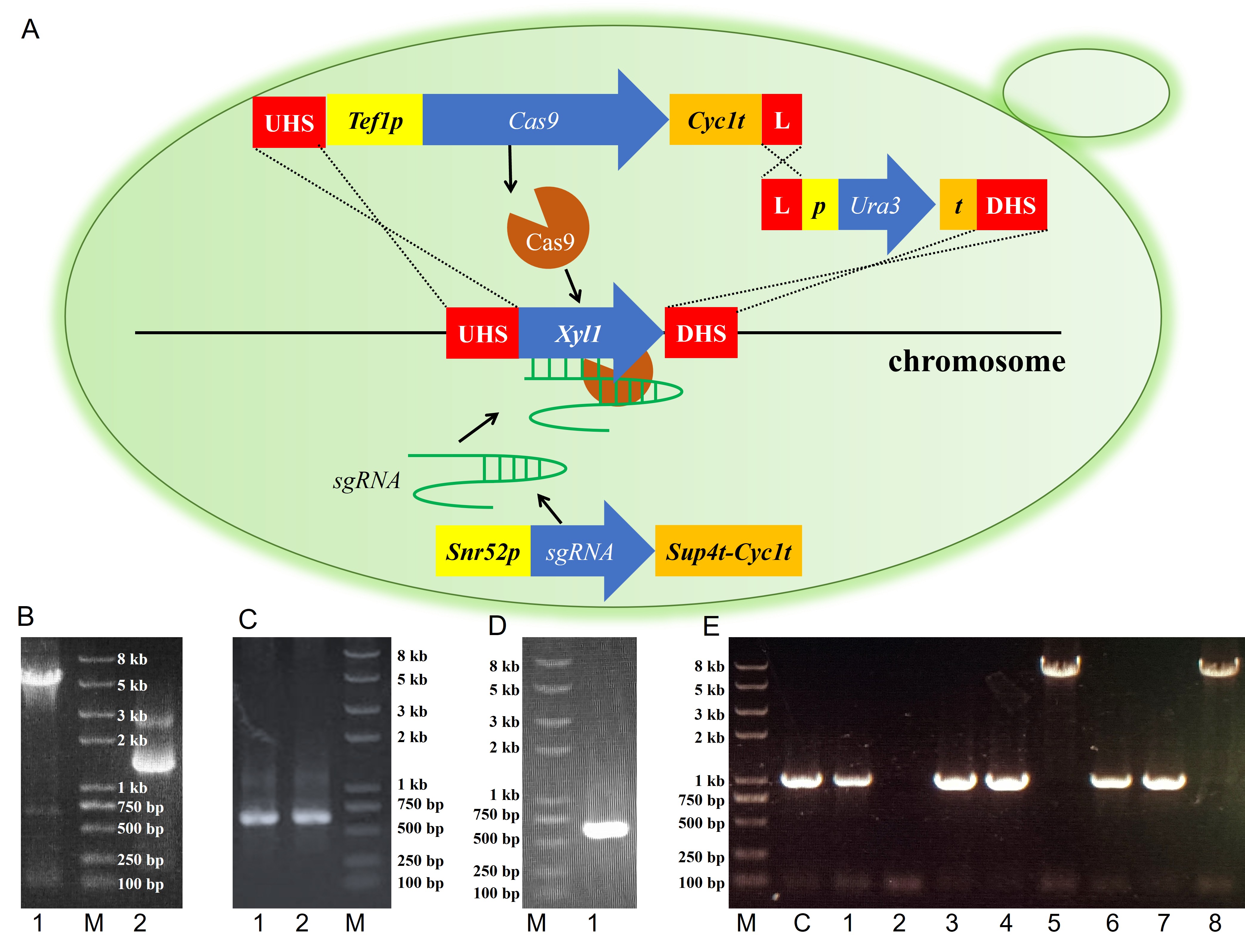


**Fig. S1** (A) The schematic diagram illustrating the integration of the Cas9 expression cassette into the genome using transient CRISPR/Cas9. (B) Amplified products of the Cas9 (line 1) and *Ura3* (line 2) expression cassettes. (C) Amplification results of the promoter and terminator regions for sgRNA construction using fusion PCR. (D) The electrophoresis results of sgRNA construction using fusion PCR. (E) The electrophoresis results of genomic PCR detection for transformant, negative amplification results are approximately 1 kb, while positive results are approximately 6 kb (lines 5 and 8).


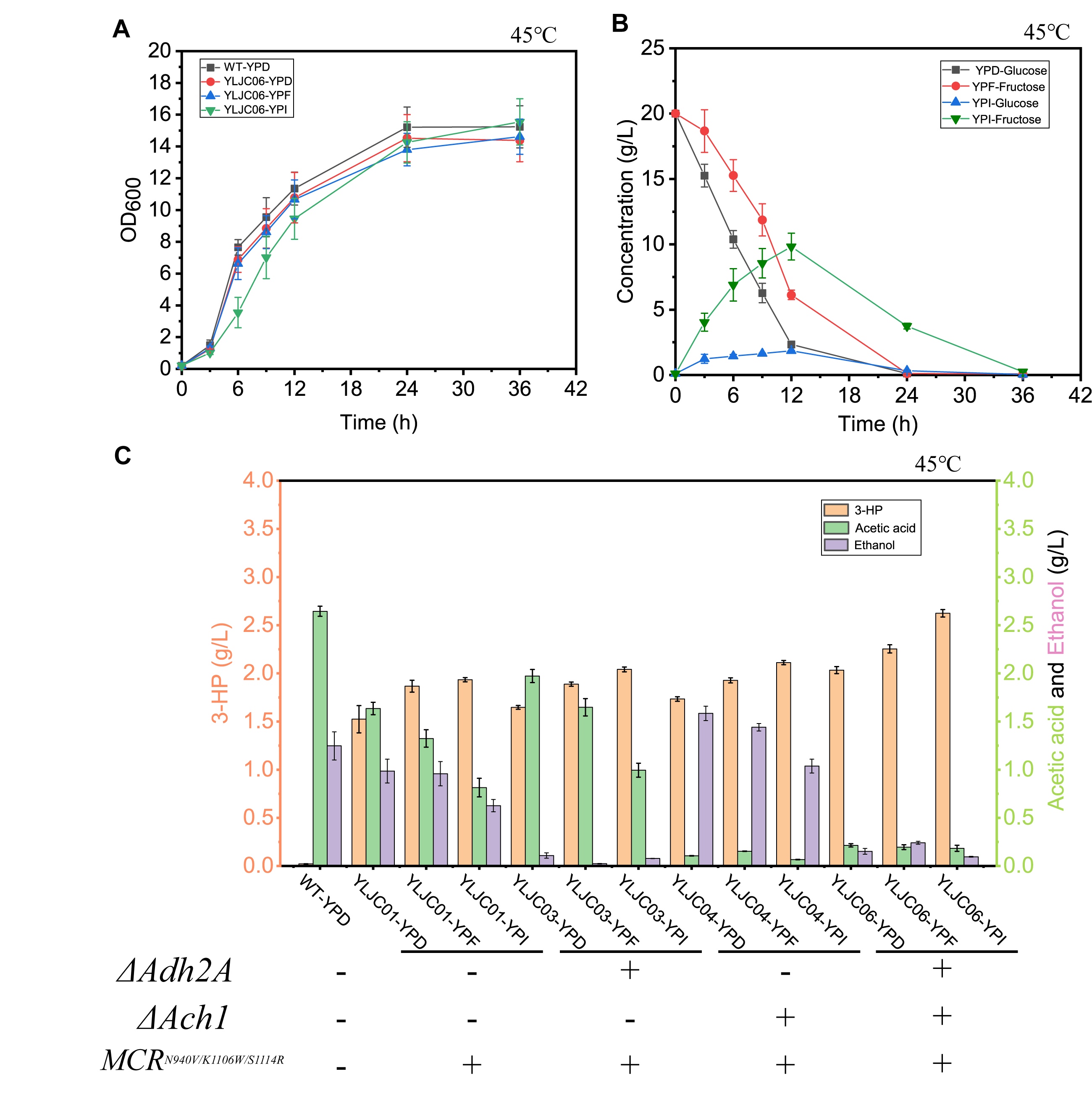


Fig. S2 The growth curve (A) and substrate consumption (A) of strain YLJC06 utilizing YPD, YPF, and YPI for the synthesis of 3-HP at 45°C. The 3-HP, acetic and ethanol concentration of WT, YLJC01, YLJC03, YLJC04, and YLJC06 fermentation with YPD, YPF, and YPI at 45^o^C.

**References**

[1]Asiatica, P., Nguyen, T.T., Lama, S., Ainala, S.K., Sankaranarayanan, M., Chauhan, A.S., Kim, J.R., Park, S. 2021. Development of *Pseudomonas asiatica* as a host for the production of 3-hydroxypropionic acid from glycerol. Bioresource Technology, 329, 124867.

[2]Avila-Cabré, S., Albiol, J., Ferrer, P. 2025. Metabolic engineering of *Komagataella phaffii* for enhanced 3-hydroxypropionic acid (3-HP) production from methanol. Journal of Biological Engineering, 19(1), 19.

[3]Borodina, I., Kildegaard, K.R., Jensen, N.B., Blicher, T.H., Maury, J., Sherstyk, S., Schneider, K., Lamosa, P., Herrgård, M.J., Rosenstand, I., Öberg, F., Forster, J., Nielsen, J. 2015. Establishing a synthetic pathway for high-level production of 3-hydroxypropionic acid in *Saccharomyces cerevisiae* via β-alanine. Metabolic Engineering, 27, 57-64.

[4]Chang, Z.S., Dai, W., Mao, Y.F., Cui, Z.Z., Zhang, Z.D., Wang, Z.W., Ma, H.W., Chen, T. 2022. Enhanced 3-Hydroxypropionic acid production from acetate *via* the malonyl-CoA Pathway in *Corynebacterium glutamicum*. Frontiers in Bioengineering and Biotechnology, 9, 808258.

[5]Chen, Z., Huang, J.H., Wu, Y., Wu, W.J., Zhang, Y., Liu, D.H. 2017. Metabolic engineering of *Corynebacterium glutamicum* for the production of 3-hydroxypropionic acid from glucose and xylose. Metabolic Engineering, 39, 151-158.

[6]Chu, H.S., Kim, Y.S., Lee, C.M., Lee, J.H., Jung, W.S., Ahn, J.H., Song, S.H., Choi, I.S., Cho, K.M. 2015. Metabolic engineering of 3-hydroxypropionic acid biosynthesis in *Escherichia coli*. Biotechnology and Bioengineering, 112(2), 356-364.

[7]Fina, A., Brêda, G.C., Pérez-Trujillo, M., Freire, D.M.G., Almeida, R.V., Albiol, J., Ferrer, P. 2021. Benchmarking recombinant *Pichia pastoris* for 3-hydroxypropionic acid production from glycerol. Microbial Biotechnology, 14(4), 1671-1682.

[8]Fina, A., Heux, S., Albiol, J., Ferrer, P. 2022. Combining metabolic engineering and multiplexed screening methods for 3-hydroxypropionic acid production in *Pichia pastoris*. Frontiers in Bioengineering and Biotechnology, 10, 942304.

[9]Jiang, X.R., Yan, X., Yu, L.P., Liu, X.Y., Chen, G.Q. 2021. Hyperproduction of 3-hydroxypropionate by *Halomonas bluephagenesis*. Nature Communications, 12(1), 1513.

[10]Kalantari, A., Chen, T., Ji, B.Y., Stancik, I.A., Ravikumar, V., Franjevic, D., Saulou-Bérion, C., Goelzer, A., Mijakovic, I. 2017. Conversion of glycerol to 3-hydroxypropanoic acid by genetically engineered *Bacillus subtilis*. Frontiers in Microbiology, 8, 638.

[11]Kang, F., Gu, F., Zhong, Y., Cui, Z., Liang, Q., Qi, Q. 2025. Expanding the genetic toolkit of Yarrowia lipolytica: Dynamic promoter engineering enables high-titer biosynthesis of 3-hydroxypropionic acid. Bioresource Technology, 432.

[12]Kildegaard, K.R., Jensen, N.B., Schneider, K., Czarnotta, E., Özdemir, E., Klein, T., Maury, J., Ebert, B.E., Christensen, H.B., Chen, Y., Kim, I.K., Herrgård, M.J., Blank, L.M., Forster, J., Nielsen, J., Borodina, I. 2016. Engineering and systems-level analysis of *Saccharomyces cerevisiae* for production of 3-hydroxypropionic acid via malonyl-CoA reductase-dependent pathway. Microbial Cell Factories, 15, 53.

[13]Kim, J.W., Ko, Y.S., Chae, T.U., Lee, S.Y. 2020. High-level production of 3-hydroxypropionic acid from glycerol as a sole carbon source using metabolically engineered. Biotechnology and Bioengineering, 117(7), 2139-2152.

[14]Kwak, S., Park, Y.C., Seo, J.H. 2013. Biosynthesis of 3-hydroxypropionic acid from glycerol in recombinant *Escherichia coli* expressing *Lactobacillus brevis dhaB* and *dhaR* gene clusters and E*. coli* K-12 *aldH*. Bioresource Technology, 135, 432-439.

[15]Lai, N.Y., Luo, Y.C., Fei, P., Hu, P., Wu, H. 2021. One stone two birds: Biosynthesis of 3-hydroxypropionic acid from CO_2_ and syngas-derived acetic acid in *Escherichia coli*. Synthetic and Systems Biotechnology, 6(3), 144-152.

[16]Li, W., Wang, T., Dong, Y.W., Li, T.X. 2020. Screening, identification, and low-energy ion modified breeding of a yeast strain producing high level of 3-hydroxypropionic acid. Microbiologyopen, 9(1), E00956.

[17]Li, Y., Wang, X., Ge, X.Z., Tian, P.F. 2016. High production of 3-hydroxypropionic acid in *Klebsiella pneumoniae* by systematic optimization of glycerol metabolism. Scientific Reports, 6, 26932.

[18]Liang, B., Sun, G.N., Wang, Z.B., Xiao, J., Yang, J.M. 2019. Production of 3-hydroxypropionate using a novel malonyl-CoA-mediated biosynthetic pathway in genetically engineered strain. Green Chemistry, 21(22), 6103-6115.

[19]Lipscomb, T., Lipscomb, M., Gill, R., Lynch, M. 2012. Metabolic Engineering of Recombinant E. coli for the Production of 3‐Hydroxypropionate. Engineering Complex Phenotypes in Industrial Strains, 185-200.

[20]Lis, A.V., Schneider, K., Weber, J., Keasling, J.D., Jensen, M.K., Klein, T. 2019. Exploring small-scale chemostats to scale up microbial processes: 3-hydroxypropionic acid production in *S. cerevisiae*. Microbial Cell Factories, 18, 50.

[21]Liu, B., Xiang, S.M., Zhao, G., Wang, B.J., Ma, Y.H., Liu, W.F., Tao, Y. 2019. Efficient production of 3-hydroxypropionate from fatty acids feedstock in *Escherichia coli*. Metabolic Engineering, 51, 121-130.

[22]Liu, S.Y., Sun, Y., Wei, T.H., Gong, D.L., Wang, Q., Zhan, Z., Song, J.Z. 2023. Engineering 3-hydroxypropionic acid production from glucose in *Yarrowia lipolytica* through malonyl-CoA pathway. Journal of Fungi, 9(5).

[23]Lu, J.F., Wang, Y.Y., Xu, M.C., Fei, Q., Gu, Y., Luo, Y.C., Wu, H. 2022. Efficient biosynthesis of 3-hydroxypropionic acid from ethanol in metabolically engineered *Escherichia coli*. Bioresource Technology, 363, 127907.

[24]Nguyen-Vo, T.P., Liang, Y.X., Sankaranarayanan, M., Seol, E., Chun, A.Y., Ashok, S., Chauhan, A.S., Kim, J.R., Park, S. 2019. Development of 3-hydroxypropionic-acid-tolerant strain of *Escherichia coli W* and role of minor global regulator. Metabolic Engineering, 53, 48-58.

[25]Nguyen, D.T.N., Lee, O.K., Lim, C., Lee, J., Na, J.G., Lee, E.Y. 2020. Metabolic engineering of type II methanotroph, *Methylosinus trichosporium* OB3b, for production of 3-hydroxypropionic acid from methane via a malonyl-CoA reductase-dependent pathway. Metabolic Engineering, 59, 142-150.

[26]Qin, N., Li, L.Y., Wan, X.Z., Ji, X., Chen, Y., Li, C.K., Liu, P., Zhang, Y.J., Yang, W.J., Jiang, J.F., Xia, J.Y., Shi, S.B., Tan, T.W., Nielsen, J., Chen, Y., Liu, Z.H. 2024. Increased CO_2_ fixation enables high carbon-yield production of 3-hydroxypropionic acid in yeast. Nature Communications, 15(1), 1591.

[27]Song, C.W., Kim, J.W., Cho, I.J., Lee, S.Y. 2016. Metabolic engineering of *Escherichia coli* for the production of 3-hydroxypropionic acid and malonic acid through β-alanine route. Acs Synthetic Biology, 5(11), 1256-1263.

[28]T., T.N., S., L., S., K.A., M., S., A., S.C., J., R.K., S., P. 2021. Development of pseudomonas asiatica as a host for the production of 3-hydroxypropionic acid from glycerol. Bioresource technology, 329, 124867.

[29]Takayama, S., Ozaki, A., Konishi, R., Otomo, C., Kishida, M., Hirata, Y., Matsumoto, T., Tanaka, T., Kondo, A. 2018. Enhancing 3-hydroxypropionic acid production in combination with sugar supply engineering by cell surface-display and metabolic engineering of *S. pombe*. Microbial Cell Factories, 17, 176.

[30]Tong, T., Tao, Z.Y., Chen, X.L., Gao, C., Liu, H., Wang, X.L., Liu, G.Q., Liu, L.M. 2021. A biosynthesis pathway for 3-hydroxypropionic acid production in genetically engineered. Green Chemistry, 23(12), 4502-4509.

[31]Wang, X.D., Hou, J.Y., Cui, J.Y., Wang, Z.W., Chen, T. 2024. Engineering *Corynebacterium glutamicum* for the efficient production of 3-hydroxypropionic acid from glucose via the β-alanine pathway. Synthetic and Systems Biotechnology, 9(4), 752-758.

[32]Wang, Y.P., Sun, T., Gao, X.Y., Shi, M.L., Wu, L.N., Chen, L., Zhang, W.W. 2016. Biosynthesis of platform chemical 3-hydroxypropionic acid (3-HP) directly from CO_2_ in *Cyanobacterium synechocystis* sp PCC 6803. Metabolic Engineering, 34, 60-70.

[33]Wu, X.Y., Cai, P., Gao, L.H., Li, Y.X., Yao, L., Zhou, Y.J. 2023. Efficient Bioproduction of 3-Hydroxypropionic Acid from Methanol by a Synthetic Yeast Cell Factory. Acs Sustainable Chemistry & Engineering, 11(16), 6445-6453.

[34]Yu, W., Cao, X., Gao, J.Q., Zhou, Y.J. 2022. Overproduction of 3-hydroxypropionate in a super yeast chassis. Bioresource Technology, 361, 127690.

[35]Zhang, Y.F., Zabed, H.M., Yun, J.H., Zhang, G.Y., Wang, Y., Qi, X.H. 2021. Notable improvement of 3-hydroxypropionic acid and 1,3-propanediol coproduction using modular coculture engineering and pathway rebalancing. Acs Sustainable Chemistry & Engineering, 9(12), 4625-4637.

[36]Zhao, L., Lin, J.P., Wang, H.L., Xie, J.L., Wei, D.Z. 2015. Development of a two-step process for production of 3-hydroxypropionic acid from glycerol using *Klebsiella pneumoniae* and *Gluconobacter oxydans* Bioprocess and Biosystems Engineering, 38(12), 2487-2495.

[37]Zhao, P., Li, Q.Y., Tian, P.F., Tan, T.W. 2021. Switching metabolic flux by engineering tryptophan operon-assisted CRISPR interference system in *Klebsiella pneumoniae*. Metabolic Engineering, 65, 30-41.

[38]Zhao, P., Ma, C.L., Xu, L.D., Tian, P.F. 2019. Exploiting tandem repetitive promoters for high-level production of 3-hydroxypropionic acid. Applied Microbiology and Biotechnology, 103(10), 4017-4031.

[39]Zhu, J.W., Xie, J.L., Wei, L.J., Lin, J.P., Zhao, L., Wei, D.Z. 2018. Identification of the enzymes responsible for 3-hydroxypropionic acid formation and their use in improving 3-hydroxypropionic acid production in *Gluconobacter oxydans* DSM 2003. Bioresource Technology, 265, 328-333.
